# Recent Advances in the Application of Chiral Phosphine Ligands in Pd-Catalysed Asymmetric Allylic Alkylation

**DOI:** 10.3390/molecules16010970

**Published:** 2011-01-21

**Authors:** Itzel Guerrero Rios, Alonso Rosas-Hernandez, Erika Martin

**Affiliations:** Departamento de Química Inorgánica, Facultad de Química, Universidad Nacional Autónoma de México, Av. Universidad 3000, 04510 México D.F., Mexico

**Keywords:** allylic alkylation, asymmetric catalysis, chiral phosphines, palladium

## Abstract

One of the most powerful approaches for the formation of simple and complex chiral molecules is the metal-catalysed asymmetric allylic alkylation. This reaction has been broadly studied with a great variety of substrates and nucleophiles under different reaction conditions and it has promoted the synthesis of new chiral ligands to be evaluated as asymmetric inductors. Although the mechanism as well as the active species equilibria are known, the performance of the catalytic system depends on the fine tuning of factors such as type of substrate, nucleophile nature, reaction medium, catalytic precursor and type of ligand used. Particularly interesting are chiral phosphines which have proved to be effective asymmetric inductors in several such reactions. The present review covers the application of phosphine-donor ligands in Pd-catalysed asymmetric allylic alkylation in the last decade.

## 1. Introduction

During the last decades, transition-metal catalysed reactions have played an important role in asymmetric organic synthesis by providing easy, selective, doable and environmentally friendly processes to produce a wide variety of organic products [1,2]. Among these, transformations resulting in the enantioselective formation of C-C bonds are particularly useful in the synthesis of complex molecules of biological and industrial interest. These processes include the recently Nobel awarded Mizoroki-Heck, Suzuki-Miyaura and Negishi cross-coupling reactions [3,4]. Asymmetric allylic alkylation (AAA) results also in C-C bond formation with the added value of furnishing optically active products [5,6,7,8,9,10,11]. Differing from cross-coupling reactions, AAA generates stereogenic centres by controlling the attack of the nucleophile to a metal-coordinated allylic substrate. 

**Scheme 1:**
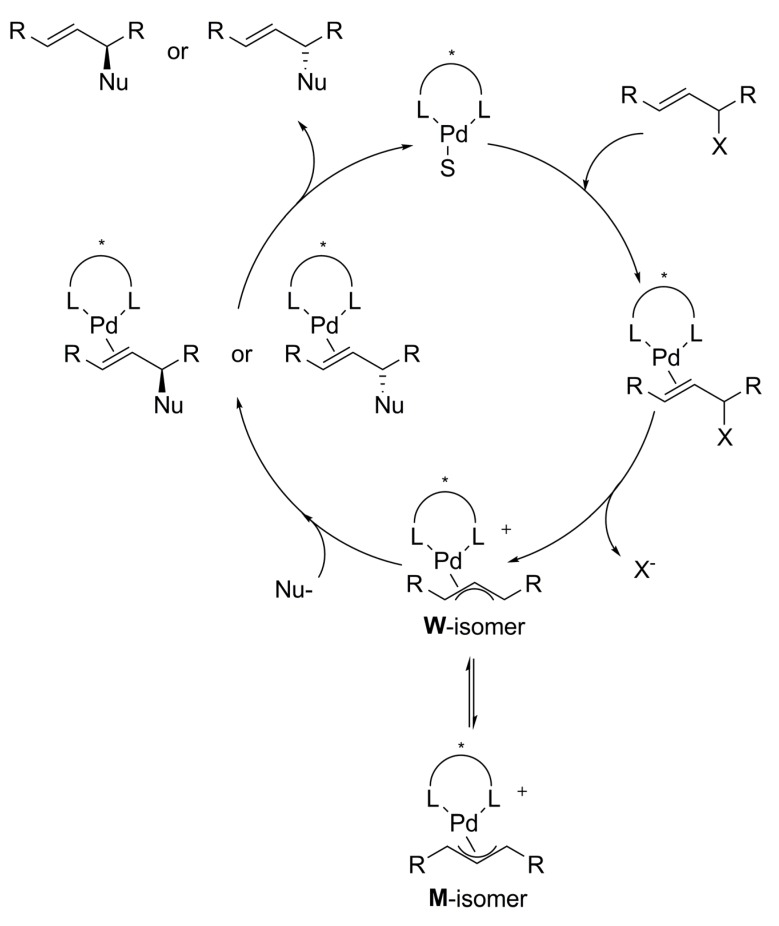
Representative catalytic cycle of palladium-catalysed allylic alkylation reaction.

The mechanism of the palladium-catalysed allylic alkylation reaction involves coordination of the allylic electrophile substrate to a low-valent metal centre followed by an oxidative addition to generate a cationic η^3^-allyl complex with the leaving group as counterion. Subsequently, attack of “soft” nucleophiles takes place on the terminal carbons of the allylic ligand at the opposite face to which the metal is bound, with concomitant reductive elimination. Alternatively, the less common “hard” nucleophiles, bind to the metal and then attack the allylic moiety. Dissociation of the resultant olefin complex regenerates the starting catalyst (Scheme 1). 

**Scheme 2:**
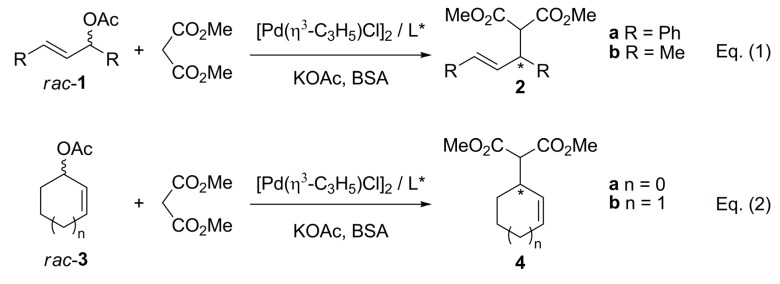
Pd- catalysed asymmetric allylic alkylation of common substrates.

Every step provides an opportunity for enantioselection depending on the experimental conditions, except for the olefin dissociation step, since the new carbon-carbon bond has already been formed. When racemic or prochiral substrates with identical substituents at the terminal allylic carbons are employed (*rac*-**1**, Scheme 2), the enantioselectivity depends on the regioselectivity of the nucleophilic attack. Moreover, regioselectivity can be controlled by the chiral ligand attached to the metal centre through stabilisation of the resultant olefin complex after the nucleophilic attack at either of the terminal allylic carbons. When a η^3^-allylmetal complex undergoes isomerisation by π−σ−π interconversions, several isomers can be formed, commonly M- and W-type isomers. Therefore, the enantioselectivity will depend on both isomer concentrations and relative rates of nucleophilic attack to each terminal allylic carbon of the isomers present in the reaction. Since many factors influence these reaction rates, a thorough control of the process is difficult. Notwithstanding, a wide variety of chiral ligands have been successfully employed, as was first demonstrated by Trost in 1977 [12]. Ever since, many catalytic systems have been proved and new chiral ligands have been specifically designed to achieve better activities and stereoselectivities. The most commonly used ligands contain phosphorous, nitrogen, sulfur or a combination of two or even three donor atoms [5,6,11,13,14,15,16].

Many transition metals can be used in the catalysed AAA (Pd, Ni, Pt, Mo, W, Rh, Ir, Ru, Cu, Fe) [2], but due to its notable efficacy, Pd is on top of the list. Additionally, this versatile reaction has been carried out with a wide variety of substrates, nucleophiles and leaving groups. The required allylic substrates contain mainly acetoxy as leaving group, although carbonates, halogens and *o*-silyl groups have been used with less success. The anionic nucleophile is made to react as its sodium salt or generated *in situ* with K_2_CO_3_, Cs_2_CO_3_, KO^t^Bu, *N,O*-bis(trimethylsillyl)-acetamide (BSA), and the recently reported ZnEt_2_. The benchmark substrates and the nucleophile commonly studied in the AAA are shown in Scheme 2. Trost alkylation conditions (i.e. *N,O*-bis(trimethylsilyl)acetamide (BSA), KOAc in catalytic amount and [Pd(η^3^-C_3_H_5_)Cl]_2_) are considered as default conditions unless otherwise stated. Other reaction conditions such as temperature, nature of the palladium precursor, or substrate and nucleophile will be defined in the text when necessary.

In the present review, we provide an overview of the asymmetric allylic alkylation reaction with a main focus on phosphine ligands in combination with palladium precursors and stabilized nucleophiles for the enantioselective formation of C-C bonds covering the last decade. 

## 2. Monophosphine Ligands

Since Trost and co-workers discovered the tremendous application of diphosphine ligands in AAA, many bidentate ligands have been designed as a main source of chiral induction. In contrast, monophosphine ligands have been developed that overcome activities and selectivities that otherwise are impossible to achieve with robust diphosphine ligands. Monophosphines can be classified in terms of their chirality: (i) central chirality on carbon or phosphorous atoms; (ii) biaryl axial chirality; and (iii) planar chirality.

### 2.1. Monophosphines with central chirality

**Figure 1:**
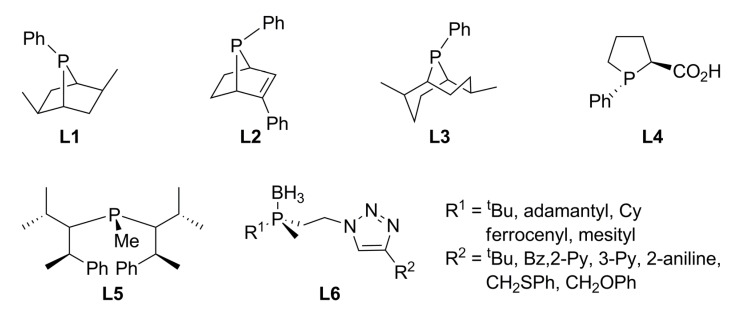
Monophosphine ligands with central chirality.

Monophosphine ligands with chirality centred on the phosphorous and/or on carbon atoms have been synthesised, with the possibility of coordination not only by phosphorous but by phosphorous-oxygen atoms in order to stabilize intermediates. Monophosphine ligands **L1**-**L4** (Figure 1) were prepared in good yields in which the phosphorous cycle is modified (by the presence of different substituents or by increasing the ring size) in order to circumvent the conformational flexibility associated with five-membered rings, known to have a negative effect on enantioselectivity, but keeping their positive effect such as control of electronic, steric an chiral properties of the ligand by addition of substituents. 

**Scheme 3:**
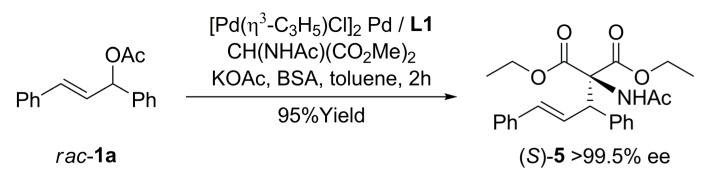
Asymmetric allylic alkylation of *rac*-**1a** with diethyl acetamidomalonate in presence of **L1**.

**L1** proved to be an effective chiral inductor in the AAA of standard biaryl linear substrate with dimethyl malonate as nucleophile [*rac*-**1a** in Equation (1), Scheme 2]. The catalytic reaction was strongly dependent on the palladium precursor used and the palladium-ligand ratio; thus, [Pd(η^3^-C_3_H_5_)Cl]_2_ as precursor in a ratio Pd:**L1** 1:2 was fundamental to reach a 99% yield and >97% ee of (*R*)-**2a**. In addition, diethyl acetamidomalonate nucleophile afforded an increased enantioselectivity (Scheme 3) [17]. 

Contrary to this result, **L2** produced 96% ee of (*S*)-**2a** in 87% yield with a Pd:**L2** ratio of 1:1, suggesting that the η^3^-allylpalladium species is stabilized by only one ligand through phosphorous and olefin coordination [18]. Crystallographic data on the complex stabilized by **L2** showed a greater *trans* influence of phosphine compared to olefin coordination, favouring the nucleophilic attack on the allylic carbon bonded *trans* to phosphorous, resulting in the (*S*)-**2a** isomer. **L3** known as 9-PBN was tested in the allylic alkylation of different cyclic substrates with CH_2_(CO_2_Me)_2_; the results are summarized in Table 1 [19]. The previously reported reaction with **L3** in combination with linear substrates *rac*-**1a** gave good results, but reaction times were as long as three days [20].

**Table 1:** Asymmetric allylic alkylation reaction with **L3**.

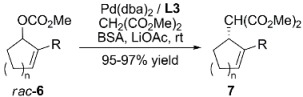	
R (n)	%ee
Ph (2); C≡C-TMS (2)	90-95
Me (1 or 2); 2-furyl (2)	50-54

In the AAA with *rac*-**1a** and CH_2_(CO_2_Me)_2_ [Equation (1), Scheme 2], **L4** produced 77% ee of (*S*)-**2a** in 86% yield. Although the authors did not comment on the results, it is possible to suggest a stabilisation of allylic intermediates through chelate species via phosphorous-oxygen coordination with the carboxylic group present in the phospholane backbone [21].

The monophosphine **L5** was prepared in good yield and stored as its BH_3_-adduct. **L5** was tested with substrate *rac*-**1a** [Equation (1), Scheme 2] varying solvent and Pd:**L5** ratios afforded 90% yield and 73% ee of (*R*)-**2a** in DMF at room temperature [22].

A family of P-chirogenic phosphine ligands **L6** containing a triazole moiety (ChiraClick ligands) was prepared in high yield allowing variations of both phosphine and triazole moieties. Study of this family in the palladium-catalysed asymmetric allylic alkylation of *rac*-**1a **in THF afforded good to quantitative conversion in most cases, while the enantioselectivity proved less satisfactory with ee’s in the range of 8-12% [23].

**Figure 2:**
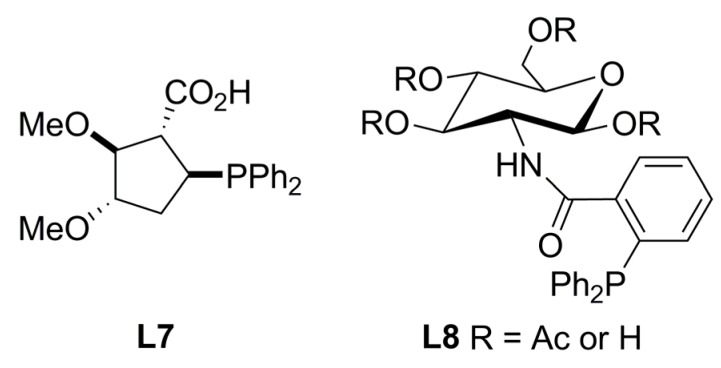
Monophosphines with central chirality on carbon atoms.

Chiral phosphinocarboxylic acid **L7** (Figure 2) was evaluated in asymmetric allylic alkylation of *rac*-**1a** [Equation (1), Scheme 2] with Pd(OAc)_2_ in THF at room temperature for 24 h affording a 93% ee of (*R*)-**2a** and 77% yield. It was suggested that **L7** forms a (*P,O*)-chelator, though no allylic palladium complex was isolated [24]. Chiral monophosphines have been synthesized with the Trost System Ligands in mind, TSL (*vide infra*), showing lower enantioselectivities. Spectroscopic analysis in solid state and in solution of the corresponding allyl-palladium complex unambiguously showed that these ligands coordinate the metal centre through P-O atoms; carboxylamide acted as the *O*-ligand. Glucosamide based ligand **L8** (Figure 2) was tested in the asymmetric allylic alkylation of *rac*-**1a** in combination with different nucleophiles. An increment of the ratio Pd:**L8** from 1:1 to 1:2 reduced the enantioselectivity from 84% ee of (*R*)-**2a** to 62% ee of (*R*)-**2a**. ES-MS analysis of the complex formed with a variety of Pd:**L8** ratios, suggested that a chelate form was accomplished in a 1:1 ratio; this species was deemed responsible for the enantioselectivity. The excess of ligand resulted in an equilibrium between two species where ligand monocoordination and chelation were achieved; this resulted in a reduced reaction enantioselectivity [25]. 

A series of ligands derived from *N*-acetylglucosamine, Naplephos (Figure in Table 2), were tested in the palladium-catalysed AAA of *rac*-**1a** with dimethyl malonate as nucleophile affording a 96% ee of (*S*)-**2a**. 

**Table 2:** Asymmetric allylic alkylation reaction with **L9**.

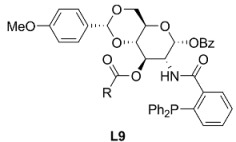	
R	(%) ee (*S*)-2a
Me	39
CH_2_Ph	85
CH_2_Cy	87
*^t^*Bu	89
(*R*)-CH(Me)Ph	91
(*S*)-CH(Me)Ph	86
CHPh_2_	91
C(Me)Ph_2_	80
CHCy_2_	80
Ph	45
CH_2_C_6_F_5_	60

Enantioselectivity was improved in the presence of bulkier ester substituents. Additionally, increasing the ratio Pd:**L9** (1:1 to 1:4) and reducing reaction time (18 h to 10 min), enhanced enantioselectivity (80 to 96% ee of (*S*)-**2a**). NMR experiments were performed to study the coordination modes of the corresponding palladium complex at different Pd:**L9** ratios. In the presence of 1 equiv. of ligand, spectroscopic analysis indicated the formation of [Pd(η^3^-C_3_H_5_)(κ-(P,O)**L9**)]^+^ species stabilised by coordination through phosphorous and oxygen of the carboxilamide group. Addition of an extra equiv. of ligand produced a new species, suggesting coordination of two phosphines **L9** in a *C_2_*-symmetrical fashion, forming the catalytic species responsible for the enhanced enantioselectivity [26]. 

### 2.2. Monophosphines with axial chirality

**Figure 3:**
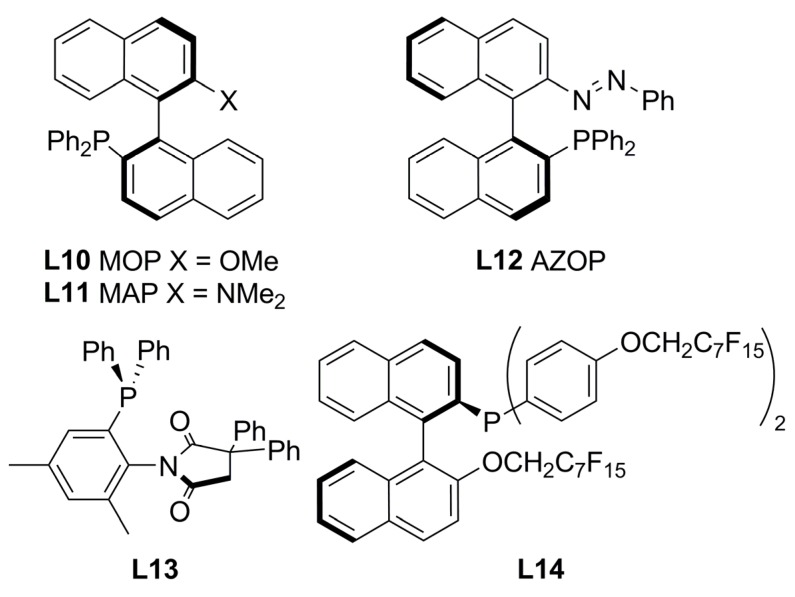
Monophosphine ligands with axial chiral.

**Scheme 4:**
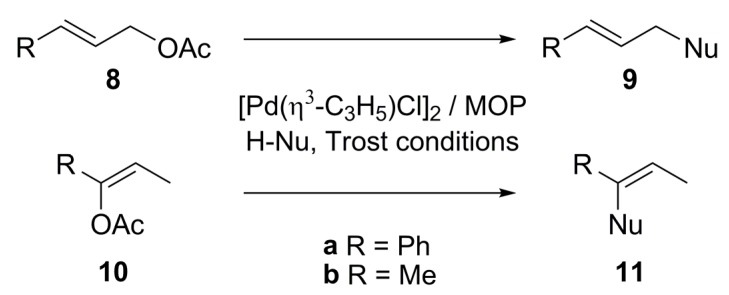
”Memory effect” with axial MOP ligands.

Monophosphine ligands derived from BINAP, MOP and MAP (**L10** and **L11**, Figure 3), described by Hayashi [27,28] and Kocovsky [29], respectively, successfully alkylated *rac***-1a** in enantiomerically pure fashion. In the case of MOP (**L10**), an interesting behaviour is observed when pure regioisomers, linear or branched phenylpropenyl acetates or cyclopentenyl esters, are alkylated: It is observed that the nucleophilic attack takes place exactly at the carbon atom previously occupied by the acetate substituent (Scheme 4) [30]. In the case of MAP (**L11**), such outcome is not observed. This behaviour is known as “memory effect” and its origins are still a matter of debate, though many studies suggest that “memory effect” is directly influenced by the modes of stabilisation of cationic species by the monophosphine ligand, which are related to the conditions employed such as concentration and nature of nucleophile, and palladium precursor [31,32].

The AZOP ligand (**L12**, Figure 3) derived from the MAP ligand (**L11**) was tested to study the effect of *trans*-*cis* photoisomerisation under UV irradiation [33]. The catalytic reaction was performed in THF at room temperature with Pd_2_(dba)_3_·CHCl_3_ as precursor, achieving 96% yield and 88% ee of (*S*)-**2a**. With MeCH(CO_2_Me)_2_ a 98% yield and 88% ee of (*S*)-enantiomer was observed; whereas, in the case of CH_2_(COMe)_2_ an 88% ee of (*S*)-product was obtained with 37% yield. The photoisomerisation effect did not affect the catalytic performance of the system, but it is interesting that while AZOP and MAP are structurally similar, these ligands behave differently in the catalysed allylic alkylation of *rac*-**1a**. When AZOP is used, (*S*)-**2a** is achieved while in the presence of MAP, (*R*)-**2a** is obtained. This means that during the catalytic process the azo group behaves like a methoxy group, as in the MOP ligand. In view of lack of evidence on how these ligands stabilise η^3^-allylpalladium intermediates, the mode of coordination of AZOP is hardly concluded. 

The structure of **L13** (Figure 3) presents axial chirality in virtue of the restricted rotation along the C-N bond. Catalytic performance in AAA of *rac*-**1a** produced modest enantioselectivities (55% ee) with low conversion (14% yield) [34]. 

As far as the improvement of cleaner technologies in which reusable catalytic systems will provide a greener alternative is concerned, it is worth mentioning the chiral “light fluorous” phosphine ligand **L14** (Figure 3) in which the introduction of long-chain perfluoroalkyl substituents increase their affinity for perfluorocarbon solvents. **L14** catalyses the AAA of *rac*-**1a** systems in benzotrifluoride achieving 99% yield with 81% ee of (*R*)-**2a**, comparable results were accomplished when the reaction was performed in toluene [88% yield and 87% ee of (R)-2a]. The reaction yields were dependent on the nature of the nucleophiles: almost quantitative yield in 1 h with 85% ee of (*R*) for CH_2_(COMe)_2_; 69% yield with 44% ee of (*S*)-product for MeCH(CO_2_Me)_2_; and 67% yield with 85% ee of (*S*)-**5** for AcNHCH(CO_2_Et)_2_ at 50°C. However, after recovery of the ligand and the corresponding palladium complex no catalytic activity was observed [35].

**Figure 4:**
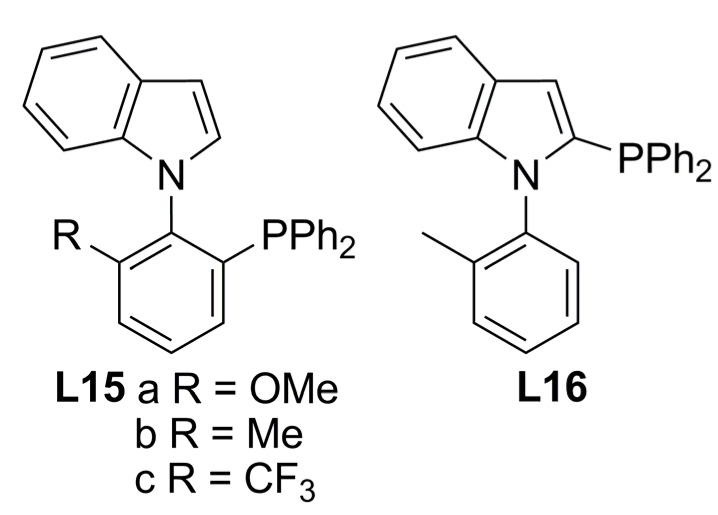
C-N bond axially chiral phosphines.

Recently, the syntheses of axially chiral phosphine ligands derived from N-aryl indoles have been described (**L15** and **L16**, Figure 4) and the function as monodentate phosphorous ligands toward Pd-allyl fragment has been proved in the case of **L15a**. 

The assessment of this chiral indole phosphine in the standard AAA [Equation (1), Scheme 2] was carried out affording excellent enantioselectivities, up to 99% ee for (*R*)-**2a**. Pd/(-)-**L15c** proved the best catalytic system (99% ee, TOF = 2h^-1^) while the Pd/**L16** system turned out less effective (50% ee, TOF = 0.06h^-1^) [36]. The solid-state structure of a π-allyl-Pd(II) complex with **L15a** and chloride showed coordination of one ligand through phosphorous. Resembling a similar structure, M or W-type intermediates can be formed prior to nucleophilic attack. In the W-type intermediate, steric hindrance between the phenyl rings of the allylic substrate and the indole moiety might be present. Therefore, the mechanism probably proceeds via M-type intermediate, and nucleophilic attack, responsible of stereoselectivity, will occur at the carbon termini *trans* to the phosphine ligand.

### 2.3. Monophosphines with planar chirality

**Figure 5:**
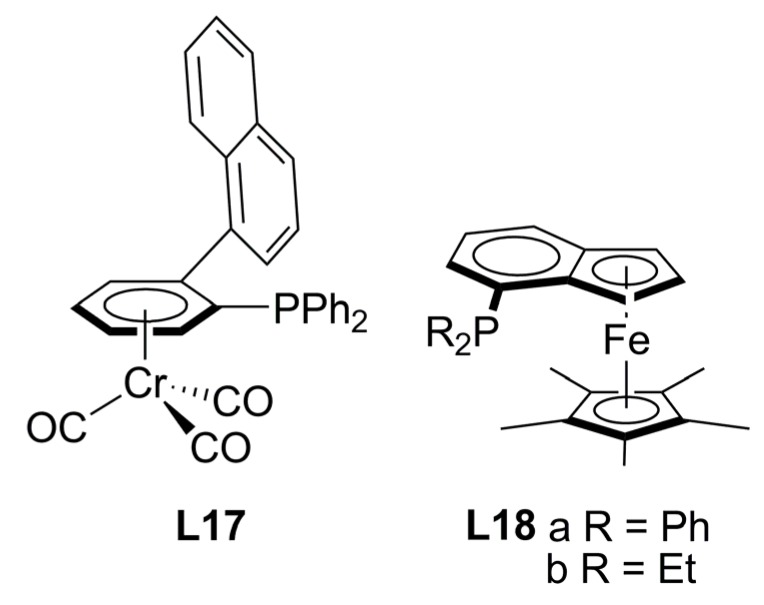
Monophosphine ligands with planar chirality.

There are scarce examples of this kind of phosphines, one example being the optically active Cr-complexed arylphosphine ligand **L17** (Figure 5). The corresponding allyl-bromide-palladium complex was tested in the AAA of *rac*-**1a**, affording 90% ee of (*S*)-**2a** and the overall reaction yielded 97% [37]. The chiral environment created by the ligand, was observed in the solid state of a π-allyl-Pd complex with only one ligand coordinated through phosphine, supporting a mechanism with a Pd(0)-monophosphine as the catalytically active species. More recently Fang and co-workers succeeded in the synthesis of a family of stable benzoferrocenyl chiral ligands (**L18**, Figure 5), each isolated in the enantiomerically pure form. In the same catalytic reaction described above and in the presence of **L18** in enantiomeric form (ratio P:Pd 2.5:1), complete conversion with moderate enantioselectivities was afforded: 39% ee of (*S*)-**2a** in 87% yield with **L18a** and up to 51% ee of (*R*)-**2a** with 93% yield with **L18b**. The authors attribute the opposite enantioselectivity induction to the catalyst conformations produced due to the monodentate nature of the ligands [38]. 

## 3. Diphosphine Ligands in Asymmetric Allylic Alkylation Reactions

The design and synthesis of chiral diphosphine ligands have played a crucial role in the development of high performance of metal-catalysed asymmetric reactions. Consequently, a great variety of chiral diphosphine ligands has been assessed in asymmetric processes. In the case of diphosphine ligands having central chirality, the Trost system ligands (TSL) are an eminent class of chiral inductors for asymmetric allylic alkylation reactions. Furthermore, they have been especially successful with allylic substrates that have proven difficult to control with other ligand systems [6,7,8,9,10,11]. Axially chiral ligands such as BINAP [39], BIHEP [40], MeO-BIHEP [41], BICHEP [42], SEGPHOS [43], SYNPHOS [44,45], TunaPHOS [46], tetraME-BITANP [47], tetra-BITIOP [48] and P-PHOS [49], have shown an excellent asymmetric induction, particularly in palladium-catalysed allylic alkylation. 

Although, planar chirality has received much less attention than the other elements of chirality (centres and axes), a wide variety of ferrocenyl ligands with planar chirality is known to date. Since the pioneering work of Ugi [50], several 1,2-disubsituted ferrocenes have been synthesised and the role of additional stereogenic centres has been discussed [28,51,52,53,54,55,56,57]. Among them, JOSIPHOS type ligands have attracted tremendous attention because their successful application in industrial synthesis of (*S*)-metolachlor, (+)-biotin and (+)-*cis*-methyldihydrojasmonate and it has stimulated intense studies in synthesis and applications of this kind of ligands [58,59,60,61,62].

### 3.1. Diphosphines with central chirality

**Figure 6:**
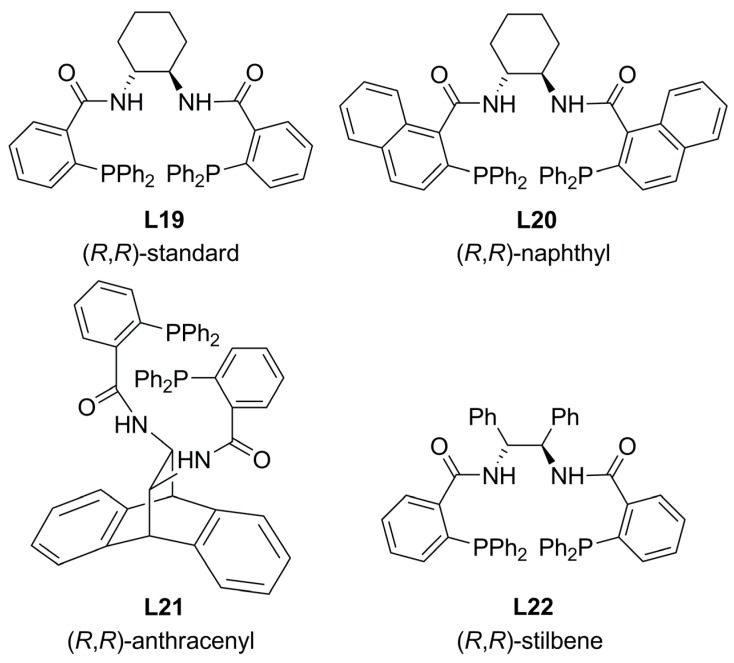
*C_2_*-symmetric diphosphine DPPBA-based ligands.

Diphosphines possessing stereogenic centres are the most popular of the chiral ligands reported to date, mainly due to their stability and outstanding ability to form highly active and selective systems in the metal-catalysed AAA. Bidentate diphenylphosphino benzoic acid (DPPBA)-based ligands (Figure 6) have shown to be an eminent class of ligands being particularly successful with allylic substrates, where other systems have failed [6,7,8,9,10].

**Figure 7:**
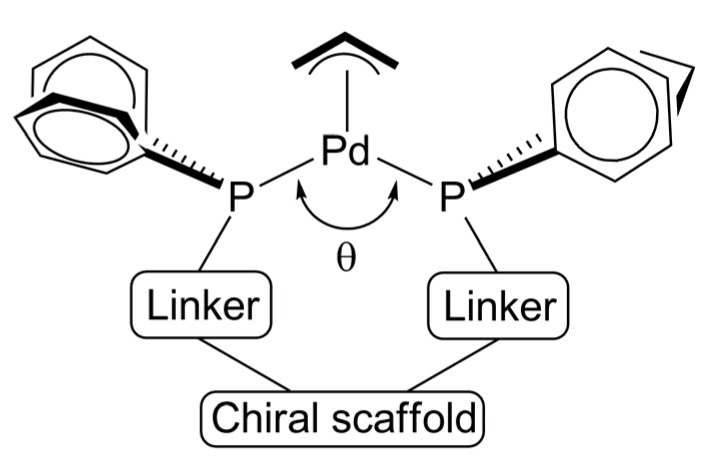
Model for ligand design.

The chiral pocket formed by Pd-ligand fragment is believed to be related to the P-Pd-P bite angle (Figure 7). Increasing the bite angle results in positioning aryl groups upward; thereby, creating a chiral space arround the η^3^-allyl moiety. Furthermore, the primary chirality of the scaffold should induce stereogenic arrangement to both diarylphosphino moieties and linkers. Based on the tremendous amount of work with DPPBA-based ligands in Pd AAA reactions, Trost’s group noticed a correlation between ligand and product stereochemistry that evolved into a mnemonic [63]. In addition, a more sophisticated working model to predict the stereochemical outcome of allylation reactions using a family of *C_2_*-symmetric DPPBA-based ligands was developed through a simplified picture model of the chiral pocket generated by the Pd-ligand fragment [64].

**Scheme 5:**
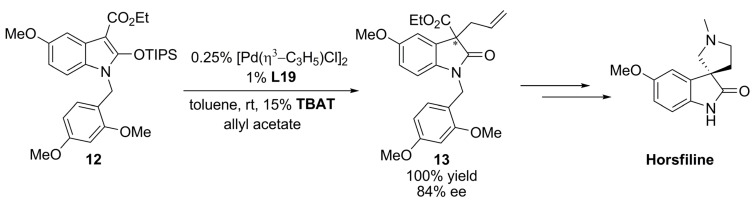
Allylation of prochiral nucleophiles in the synthesis of horsfiline.

The allylation of prochiral nucleophiles has been applied to the synthesis of natural products [65]. Using **L19 **as chiral inductor, it was possible to obtain a key intermediate (**13 **in Scheme 5) of the total synthesis of the oxindole alkaloid horsfiline showing analgesic properties with excellent yield and high enantioselectivity. The catalytic reaction is carried out with tetrabutylammonium difluorotriphenyl-silicate (TBAT) in order to generate the enolate anion.

**Scheme 6:**
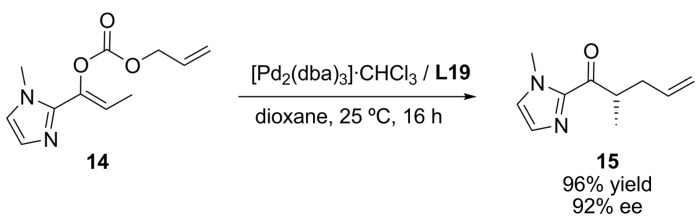
Decarboxylative allylic alkylation reaction.

Recent reports on decarboxylative allylic alkylation reaction of allyl-enol carbonates have provided a feasible means to perform enantioselective carbon-carbon bond formation [66]. In the case of the 2-acylimidazole-derived enol carbonate **14**, it was possible to synthesise highly enantioenriched 2-acylimidazole **15** with the chiral ligand **L19** (Scheme 6). The enantioenriched 2-acylimidazole product **15** can be easily converted in the corresponding carboxylic acid, ester, amide, and ketone derivatives with complete retention of enantiopurity.

**Figure 8:**
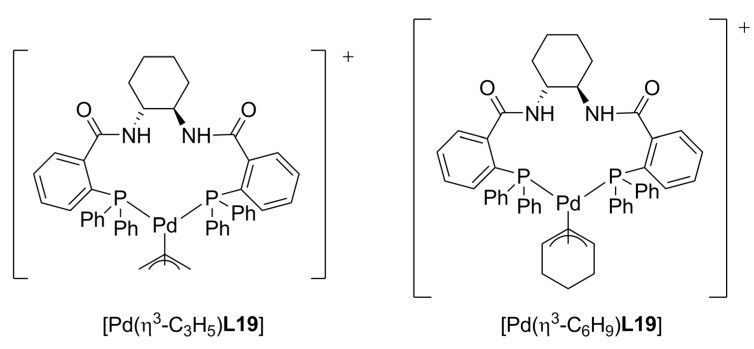
Cationic complexes of the (*R*,*R*)-standard ligand studied by NMR.

In an attempt to develop a new model to study the enantioselectivity in the allylic alkylation of cyclohexenyl derivatives **3b **[Equation (2), Scheme 2] using **L19**, a combined computational and ^2^H/^13^C label-NMR experiments of the corresponding η^3^-allyl and η^3^-cyclohexenyl complexes (Figure 8) have been carried out [67]. Although the model proposed by Trost has wide application and can be used to rationalise the outcome from nearly all of the optimized reactions catalysed by the system Pd/**L19** and related TSL ligands [64], detailed information about the intermediates involved in the reactions would allow a structural basis for rationalization of the selectivity, and would facilitate further development and application of the TSL class. It was found that H-bonding interaction between the enolate oxygen of the malonate and the amide on the concave surface of fragment Pd/**L19**, is responsible for selectively directing the enolate carbon to the proximal (*pro*-*S*) terminus of the η^3^-C_6_H_9_ unit. This interaction provides not only a new rationale for the observed selectivities but also a catalyst design feature for further related reactions.

**Scheme 7:**
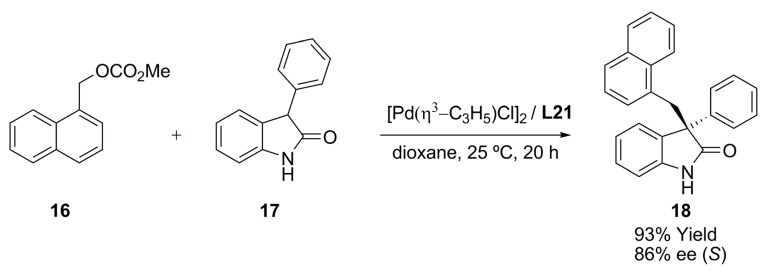
Asymmetric benzylic alkylation reaction.

Recent reports by Trost deal with palladium-catalysed asymmetric alkylation of benzylic methyl carbonates with a variety of unprotected 3-aryl oxindoles as prochiral nucleophiles (Scheme 7) [68]. 

When unprotected 3-phenyl oxindole **17** was employed as nucleophile in combination with **L21** as chiral ligand, it was possible to obtain product **18** with high enantioselectivity. It was noticed that decreasing the reaction temperature was detrimental for activity, and mildly beneficial for enantioselectivity. Other chiral diphosphine ligands (**L19**, **L20**, **L22**, Figure 6) exhibited similar activity but lower enantioselectivity. The presence of an electron-donating or electron-withdrawing group at the *para*-position of the 3-phenyl moiety of **17** did not significantly affect the reaction enantioselectivity. Finally, introduction of a benzylic group at the 3-position of oxindoles via asymmetric benzylic alkylation in both, high yield 98% and high enantiomeric excess 96%, makes this reaction a remarkable tool in organic synthesis.

**Figure 9:**
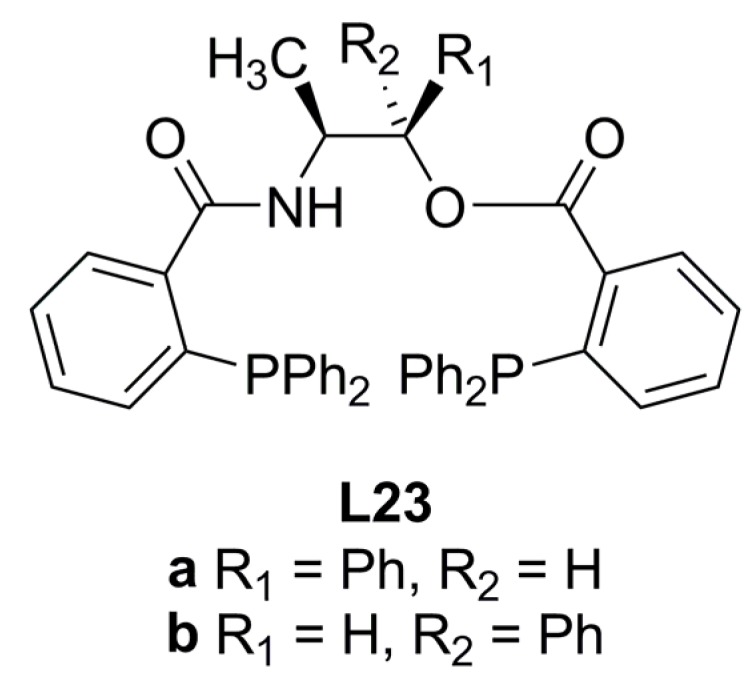
Ephedrine derivated ligands.

Mahadik reported the use of (1*R*,2*S*)-norephedrine and (1*S*,2*S*)-seudonorephedrine as chiral scaffold for the construction of ligands **L23a** and **L23b **(Figure 9) [69]. These compounds were employed in the asymmetric allylic alkylation reaction shown in Equation (1), Scheme 2. It was determined that these ligands **L23a** and **L23b** afforded (*S*)-**2a** with 88% and 63% ee, respectively. In addition, it was observed that the level of enantioselection of the alkylation reaction using **L23a** did not vary when the amount of the palladium precursor was changed.

**Figure 10:**
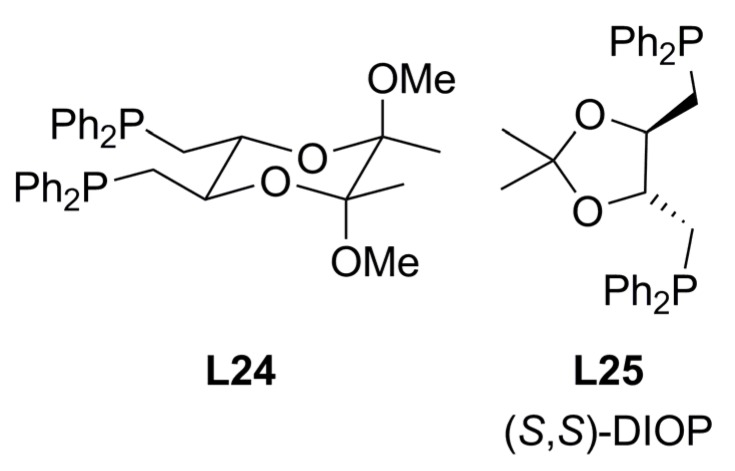
Berens’ DIOP analogue ligands.

An inexpensive chiral ligand **L24 **related to DIOP **L25 **(Figure 10) can easily be accessed from (+)-tartaric acid, as reported by Burke *et al* [70]*.* Using **L24** in the reactions depicted in Equation (1) and (2) (Scheme 2) with a variety of Pd precursors, solvents and other reaction conditions afforded moderate to good enantioselectivities (max. 60% ee (*R*)-**2a**). However, ligand **L24** was inherently superior to **L25** with regard to enantiofacial discrimination. Varying the nature of the precursor from [Pd(η^3^-C_3_H_5_)Cl]_2_ to [PdCl_2_(CH_3_CN)_2_], Pd(OAc)_2_ or [Pd_2_(dba)_3_]•CHCl_3_, led to no significant differences in ee and lower conversions. In the case of the alkylation of *rac-***3b**, the overall results were inferior to those using *rac-***1a **with a maximum conversion of 27% using THF. For this substrate, there was little difference in the enantiodiscrimination induced by either ligand **L24** or **L25**.

**Figure 11:**
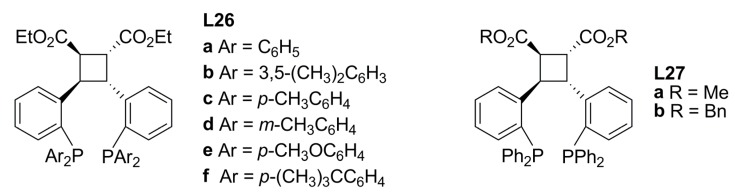
Diphosphines containing a cyclobutene backbone.

A new type of *C_2_*-symmetric diphosphine ligands (**L26a**-**f**, **L27a**-**b** in Figure 11) with a cyclobutane backbone, which can be considered as analogues of **L19-L22**, have been tested in the Pd-catalysed allylic substitution using linear substrate *rac-***1a **and cyclic substrate *rac-***3b** [Equation (1) and (2), Scheme 2] [71]. **L26a**-**f** showed excellent asymmetric induction in the reaction with linear substrates to give the corresponding allylation products in 91-99% yields with 96-99% ee. The extension of the catalyst systems to the allylic substitution of *rac-***3b** demonstrated that the reaction smoothly proceed to give the corresponding cyclic allylation product **4b** in good yields (70-80%) and enantioselectivities (78-87%). In order to explore the relationship between the carboxylate groups on the cyclobutane backbone of the diphosphine ligands and the enantioselectivity of the reaction, ligands **L27a**-**b** were synthesised. Comparing the enantioselectivities obtained in the reactions of Scheme 2 for ligands **L26a**, **L27a** and **L27b**, it was concluded that selectivity dramatically decreased with increasing the size of the ester groups. When the bisphosphine ligand **L27b**, which contains benzyl ester groups on the cyclobutane backbone, was used, the enantioselectivities of the reaction dropped to 80% ee.

One of the problems of homogenous asymmetric catalysis is the separation and recycling of the catalyst. To overcome this situation, the immobilization of catalysts on soluble polymer supports has received considerable attention. Owing to the high order of asymmetric induction of ligands **L26a**-**f** and **L27a**-**b** in Pd-catalysed allylic substitution reactions, a new type of soluble polymer-supported (PEG-OMe) diphosphine ligand was prepared [72]. The performance of the Pd complex of the MeO-PEG-supported ligand in allylic substitution reaction with *rac-***1a** is outstanding (>99% yield and 96.4% ee.). In particular, the recovered catalyst could be reused nine times with high enantioselectivities (89.5–97.2% ee).

**Figure 12:**
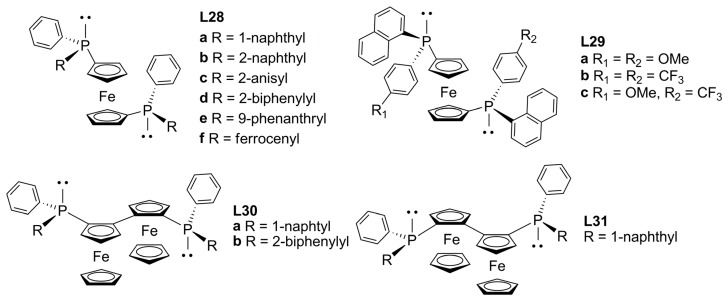
P-Stereogenic ferrocenyl diphosphines.

Another important class of diphosphines with central chirality is P-chiral diphosphine ligands based on dppf (1,1′-bis-(diphenylphosphino)ferrocene). Bearing different aryl substituents on phosphorous, these ligands were found to induce high enantioselectivities in rhodium-catalysed asymmetric hydrogenations of cinnamic acid derivatives [73]. Steric effects imposed by the nature of the substituents on chiral phosphorous and in the backbone (bite angle variation) and the influence of electronic properties on the ligand were studied by the use of phosphorous-stereogenic *C_2_*-symmetrical ferrocenyl and bisferrocenyl diphosphines (**L28**-**L31**, Figure 12) [74]. 

The performance of this group of ligands in the allylic alkylation of *rac-***1a** [Equation (1), Scheme 2], with reaction times from 2 to 20 h at room temperature, yielded more than 80% of alkylated product. **L28a**-**f** ligands differ from each other by the different sizes of the respective aryl substituents on phosphorous, as a consequence, the enantiodiscrimination varied considerably.

Ligand **L28b**, bearing 2-naphthyl moieties, showed a poor performance, which might be rationalized by its resemblance with the achiral dppf chelating ligand. Consequently, substitution in *orto*-position of the aryl group appeared crucial, but not sufficient for good asymmetric induction. The best results within this class of dppf-analogues were obtained employing diphosphine **L28f** (81% ee). In the case of the alkylation of substrates *rac-***1b** and *rac-***3b**, the performance of the catalysts was unsatisfactory since low enantiomeric excesses were achieved with moderate activities. Solid state analysis of the crystal structure of complex [Pd(1,3-diphenyl-η^3^-allyl)(**L28a**)]BF_4_ revealed a marked distortion of the η^3^-allyl fragment and, therefore, the site of nucleophilic attack is imposed by a combination of steric constraints and π-π interactions. Bulky allyls as well as proper ligand substituents are required to induce useful levels of enantiodiscrimination.

**Figure 13:**
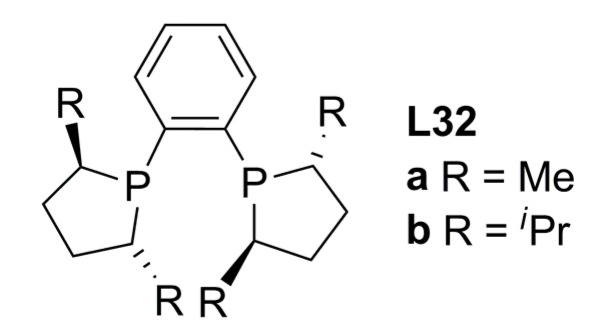
DuPHOS ligands.

Recently, Burke and co-workers carried out a comparison of (*R*,*R*)-Me-DuPHOS **L32a** and (*R*,*R*)-*^i^*Pr -DuPHOS **L32b **(Figure 13) ligands in the palladium-catalysed allylic alkylation reaction shown in Scheme 2 [75]. 

Excellent enantioselectivities (up to 98%) were obtained with **L32b** though substrate conversions were moderate in all cases (only up to 60%). It was assumed that this may be related to the considerable steric hindrance inherent in the active η^3^-allyl-palladium species formed prior to nucleophilic attack. In all cases the (*R*)-enantiomer of the alkylated malonate product was the major isomer, surprisingly different to the results obtained using **L32a** [76]. On the basis of computational and kinetic studies it was concluded that the steric hindrance generated during the malonate nucleophilic attack step might be the reason for the configurational switch between the two systems. Also, slower reaction rates in the formation of η^3^-1,3-diphenylallyl-palladium complex with **L32b**, explains the low activities of this ligand compared to **L32a**.

**Figure 14:**
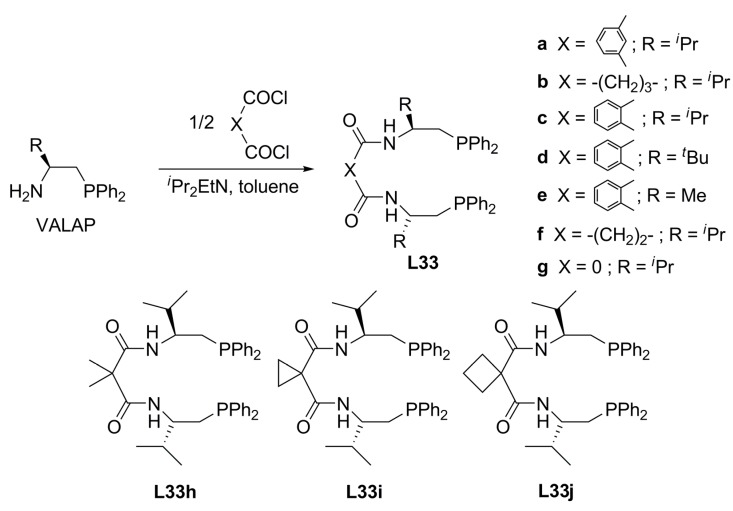
*L-*Valine-based hybrid diphosphines.

Morimoto and co-workers have reported *C_2_*-symmetric diamide-linked diphosphine ligands based on the amide–phosphine hybrid ligand VALAP which could be readily derived from *L*-valine (Figure 14) [77]. The versatile system consisting of the hetero-hybrid types or the diphosphines has actually played an important role in asymmetric allylic alkylation with diverse substrates and nucleophiles. 

**Scheme 8:**
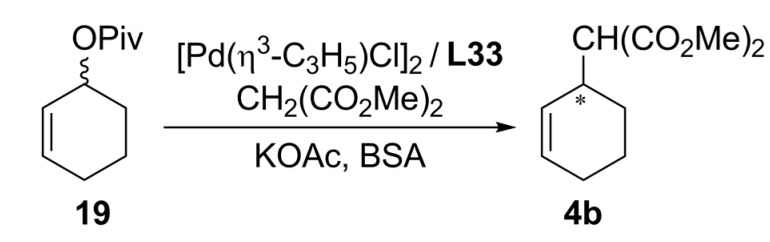
Asymetric allylic alkylation using VALAP type ligands.

Due to the characteristics of the chiral diphosphine ligands with *C_2_*-symmetry elements possessing a large bite angle remarkably high enantioselectivity is induced for sterically less demanding cycloalkenyl substrates. Diphosphine ligands **L33a**-**c **and **L33f **were examined in palladium-catalysed asymmetric allylic substitution of cyclohexen-2-yl pivalate **19 **(Scheme 8). Surprisingly, **L33c **exhibited a high level of asymmetric induction affording 99% ee (*S*)-**4b **and 64% yield. On the other hand, ligands **L33a** and **L33f** did not display any catalytic activity. Unfavourable complexation of the ligands and obstruction in the oxidative addition of the Pd(0) complex or a nucleophilic attack to the π-allyl complex by steric effects of the ligands were suggested as possible causes of the observed behaviour.

Since diphosphine ligand **L33c** afforded high levels of asymmetric induction in palladium-catalysed enantioselective alkylations of cyclic allyl substrates, the catalytic performance for 1,3-diphenyl-2-propenyl pivalate **20** as linear allyl substrate was also examined (Table 3) [78]. 

**Table 3:** Catalytic behaviour of ligands **L33** in asymmetric allylic alkylation of **20**.

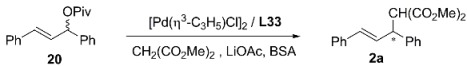			
Entry	Ligand	Yield (%)^b^	ee (%)^c^
1	**L33a**	13	64 (*R*)
2	**L33b**	5	83 (*R*)
3	**L33c**	14	72 (*S*)
4	**L33d**	52	81 (*S*)
5	**L33e**	40	62 (*S*)
6	**L33f**	4	47 (*R*)
7	**L33g**	3	5 (*S*)
8	**L33h**	54	84 (*S*)
9	**L33i**	45	87 (*S*)
10	**L33j**	50	93 (*S*)

^a^ Molar ratio: [Pd(η^3^-C_3_H_5_)Cl]_2_ / **L33**/ **20 **/ CH_2_(CO_2_Me)_2_ / BSA / AcOLi = 2.5 / 6 / 100 / 300 / 300 / 5. ^b^ Isolated yield by preparative TLC on silica gel. ^c^ The enantiomeric excess was determined by HPLC with a chiral column, Daicel Chiralpack AD.

When the alkylated linker was modified by increasing the number of carbons, **L33b** and **L33f**, it was possible to increase ee values [47-83% ee of (R)-2a]; absence of carbons, **L33g**, afforded 5% ee of (*S*)-**2a**. 1,2- and 1,3-aryl linkers gave different enantiomers in low yield (entries 1 and 3 in Table 3, respectively). Voluminous R groups increased the yield together with ee [40-52% yield, 62-81% ee of (S)-2a]. The results are improved when the rigidity of ligand was modified, **L33h-j** [45-54% yield and 84-93% ee of (S)-2a]. Summarising, it was observed that the tendency was different in the reactions using acyclic and cyclic substrates.

### 3.2. Diphosphines with axial chirality

During the 90’s decade, much effort was done on the screening of axially chiral diphosphines in asymmetric catalytic reactions, with BINAP the most investigated ligand. As a result, several structural modifications on the biaryl skeleton have been developed in order to improve enantioselectivity in asymmetric catalytic reactions. Among the most relevant modifications are variation of aryl groups on phosphorous atoms [79] and structural changes in the atropoisomeric biaryls such as H_8_-BINAP, MeO-BIHEP, BIHEMP and SEGPHOS [80]. 

**Figure 15:**
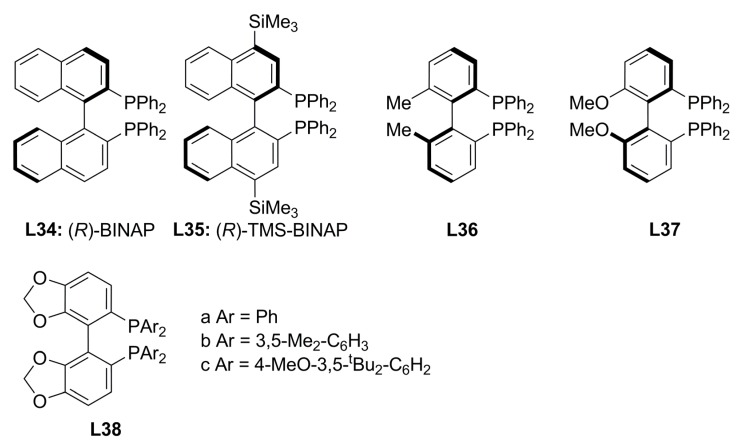
Main atropoisomeric diphosphines.

Kawano and Ito reported the first successful application of Pd/**L34 **catalytic system in the allylations of α-acetamido-β-ketoesters, **21**, with 3-substituted 2-propenyl acetates, **8**, at -30 °C in toluene, obtaining alkylated products **22a-d **with 76-95% ee in good yields (see Table 4). Based on the X-ray crystal structure of [Pd(η^3^-C_3_H_5_){(*R*)-BINAP}]ClO_4_, it is proposed that the phenyl groups of BINAP ligand may interact with the prochiral nucleophile favouring the attack by one of the enolate faces resulting in the observed enantioselectivity. For allylic substrates **8c** and **8d**, the nucleophilic attack discrimination of the allylic carbons is enhanced by the steric hindrance of phenyl allylic substituent. This high regioselectivity leads to a better enantioface-selection of the enolate, improving the ee value. Optically active α-allyl-α-acetamido-β-ketoesters **22**, are useful precursors for the synthesis of β-hydroxy-α-alkyl-α-amino acids [81]. 

Later, a series of BINAP-derivatives with sterically demanding substituents at 4 and 4’ positions of binaphthyl skeleton were developed [82,83] and evaluated in asymmetric carbon-carbon bond formation [84]. Substitutions by trimethylsylil groups (**L35**) showed a positive effect on the enantioselectivity of the allylations of **21** (Table 4). In addition, enhancement of asymmetric induction was observed when the steric effect of the substituents at both, cinnamyl acetate and nucleophile was increased. In the solid state, η^3^-allyl-palladium stabilized with **L34** and **L35** respectively, showed that the dihedral angle between the naphthyl rings is smaller when SiMe_3_ substituents are present (76.7° for **L35** and 79.9° for **L34**). The small dihedral angle enhances the steric hindrance between phenyl groups and the coordinating η^3^-allyl moiety, leading to a better stereodiscrimination in the nucleophilic attack on the η^3^-allyl group.

**Table 4:** Asymmetric allylation of α-acetamido-β-ketoesters.^a^

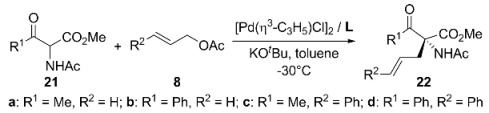				
Ligand	Product	Time, h	Yield, %	ee, %
**L34**	**22a**	24	84	76
**L34^b^**	**22a**	24	87	68
**L35^b^**	**22a**	24	75	77
**L34**	**22b**	24	92	80
**L34^b^**	**22b**	24	93	72
**L35^b^**	**22b**	24	90	84
**L34**	**22c**	2	87	94
**L34^b^**	**22c**	24	78	90
**L35^b^**	**22c**	24	68	93
**L34**	**22d**	48	71	95

^a ^The ratio 1:2: KO*^t^*Bu:Pd:**L** was 100:150:120:0.5:1.05. ^b^-25°C (taken from Ref. [84]).

Moreover, **L35** gave better enantioselectivities than unsubstituted **L34** when dimethyl malonate sodium salt or dimethyl acetamidomalonate cesium salt were used as nucleophiles in the alkylation of *rac*-**1a**. Pd/**L34 **catalytic system gave the alkylated product in 25% ee of (*R*)-**2a**, while **L35 **afforded 80% ee under the same conditions. In the case of dimethyl acetamidomalonate the effect on the asymmetric induction was less dramatic, 84% and 94% of ee was obtained employing **L34** and **L35**, respectively. A similar behaviour was observed in the AAA of cyclohexenyl acetate [*rac*-**3b**, Equation (2), Scheme 2] with dimethyl malonate sodium salt [Pd/**L34** 40% ee of (*S*)-**4b** and Pd/**L35** 57% ee of (*S*)-**4b**].

Previous studies indicated that (*S*)-BINAP was an effective chiral inductor in the AAA of *rac*-**1a** with dimethyl acetoamidomalonate as nucleophile [94% ee of (S)-2a] [85]. In contrast, when (*S*)-Binap was used with dimethyl malonate, the enantioselectivity strongly depends on the reaction conditions, 34% ee of (*R*)-**2a** when sodium salt was used, while 90% ee of (*R*)-**2a** was attained employing BSA as base [86].

**Scheme 9:**
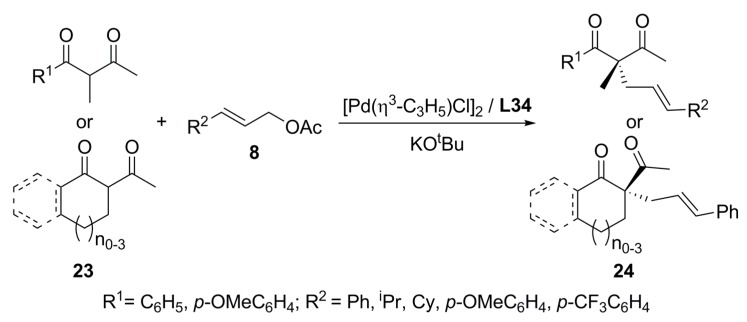
Asymmetric allylation of 1,3-diketones.

Furthermore, Pd/**L34** system has been tested in the catalytic asymmetric allylation of 1,3-diketones, **23**, providing 2,2-dialkyl-1,3-diketones **24** with 64-89% ee in good yields (Scheme 9). Asymmetric induction mainly depends on the γ-substituent nature of the allylic substrates. When cinnamyl acetate was used several unsymmetrical 1,3-diketones were alkylated affording good enantioselectivities (77-89% ee). Then, BINAP has shown again that is an effective chiral inductor for the formation of quaternary carbons [87]. 

**Scheme 10:**
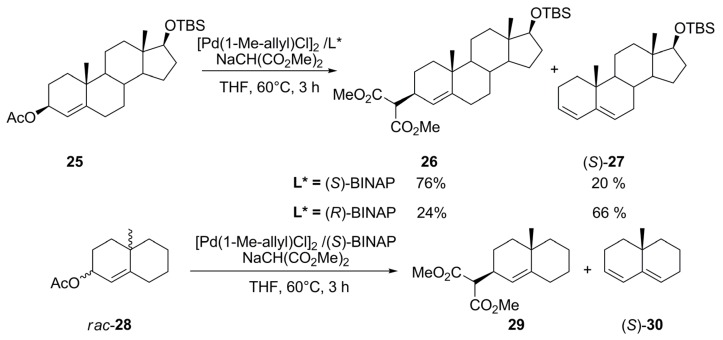
Asymmetric allylic alkylation and H-abstraction by nucleophile.

Depending on the configuration of BINAP it was possible to lead the reaction preferentially toward either the nucleophilic or the elimination product in a palladium-catalysed reaction of optically active bicyclic compounds, employing [Pd(1-Me-Allyl)Cl]_2_/BINAP under AAA conditions. As shown in Scheme 10, the reaction of (2*S*,4a*S*)-**25** and the malonate anion catalysed by Pd/(*S*)-BINAP catalyst gave (2*S*,4a*S*)-**26** in 76% yield with the diene product [(*S*)-**27** in 20% yield] as a minor product, whereas, the reaction using Pd/(*R*)-BINAP under similar conditions afforded the diene [(*S*)-**27** in 66% yield] as major product and only 24% yield of alkylated product (2*S*,4a*S*)-**26**. 

Consequently, the reaction between *rac*-**28** and Pd/(*S*)-BINAP proceeded enantio-distinctively favouring the alkylation pathway from substrate (2*S*,4a*S*)-**28**, and elimination product (*S*)-**30** from (2*R*,4a*R*)-**28**. The effect of the amount of nucleophile on kinetic resolution of substrate was also observed, which allowed to conclude that malonate anion acts as nucleophile for alkylation and as base for elimination at different rates, alkylation being faster than elimination [88]. Since the nucleophilic attack at the quaternary carbon is sterically hindered, H-abstraction reaction becomes feasible. Therefore, the chemoselectivity of this reaction depends on the relative rates of the nucleophilic attack (formation of **29**) and H-abstraction (formation of **30**).

**Figure 16:**
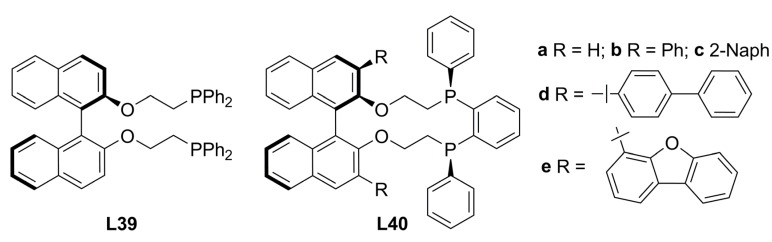
High-membered diphosphines.

New modifications on (*R*)-1,1’-binaphtol were carried out to produce non-cyclic **L39 **and macrocyclic **L40** chiral diphosphines bearing axial chirality (Figure 16) and were tested in the classical palladium-catalysed asymmetric allylic alkylation reactions [Equation (1), Scheme 2]. 2,2´-and 3,3’-susbtitutions on 1,1’-binaphtyl backbone were done in order to increase the steric interaction between ligand and substrate in palladium active species. In the case of reaction with *rac-***1a**, the alkylated product (*S*)-**2a** was obtained in high yields with 98-86% ee employing **L40** under Trost’s basic conditions while the non-cyclic ligand **L39**, gave lower ee’s (70-66%). **L40e** was the best chiral inductor in this case. The enantioselectivity strongly depends on the substrate nature since high enantioselectivities were obtained with *rac-***1a** whereas for *rac-***1b** and cyclic substrates *rac-***3**, substrates with minor steric hindrance, the asymmetric induction was very low [89]. 

**Scheme 11:**
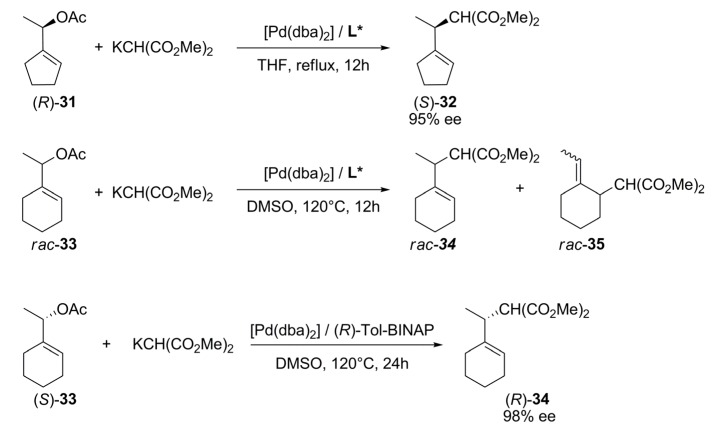
Asymmetric alkylation of cyclic substrates.

Cycloalkenyl acetates, **31** and **33** in Scheme 11, were obtained via kinetic resolution by enzymatic acylation and afterward were alkylated with dimethyl malonate using Pd/diphosphine catalysts in an attempt to study the transference of chiral information from an enantioenriched substrate to the product. The alkylation of cyclopentenyl acetate **31** was regio- and stereo-selective providing >95% ee of (*S*)-**32** for all assayed ligands [non-chiral dppe, centrally chiral (*S*,*S*)- or (*R*,*R*)-DIOP, (*R*)-BINAP **L34**]. As expected, for the alkylation of the *rac*-**31** substrate using these ligands, no enantiomeric excess was observed. Under these conditions, neither the intermediate η^3^-allyl palladium complex epimerizes nor the substrate (*S*)-**31** undergoes racemization. Whereas the alkylation of **31** is highly regioselective (95:5), the regioselectivity in the case of **33** was unsuccessfully controlled under the same conditions. However, (*R*)-tol-BINAP proved to be an effective chiral ligand to produce a single regioisomer from (*S*)-**33** with >99% ee of (*R*)-**34** but two regioisomers from (*R*)-**33**. Products **34 **and **35** are formed by nucleophilic attack to each terminal allylic carbons thus, the regioselectivity of the attack controls the formation of these isomers. When the nucleophilic attack takes place at the allylic carbon of the ring, **35** is formed, and when the nucleophile attacks the allylic carbon of the linear fragment, **34** is produced. Therefore, the regio- and enantioselectivity in the alkylation of cyclohexenyl acetates, could be controlled by the proper choice of stereochemistry of both, enantiopure substrate and catalyst [90]. 

The introduction of two non equivalent diarylphosphanyl substituents into the 2- and 2’-positions of 1,1’-binaphthyl has been successfully accomplished from (*S*)-BINOL to obtain chiral diphosphine ligands with non equivalent phosphorous atoms. This strategy was followed previously to reach an electronic or steric discrimination via ligand donor atoms as in the case of high efficient chiral controller JOSIPHOS (**L45**, Figure 19) [91]. Ligands (*S*)-**L41a-b** were compared with (*S*)*-*BINAP in the Pd-catalysed allylic alkylation of *rac*-**1a** with dimethyl malonate under Trost’s basic conditions. The *o*-tolyl-substituted diphosphine (*S*)-**L41b** [85% ee (R)-2a] was a better chiral inductor than both, *p*-tolyl-susbtituted diphospine (*S*)-**L41a** and (*S*)-BINAP, 32% and 34% ee of (*R*)-**2a**, respectively [92].

**Figure 17:**
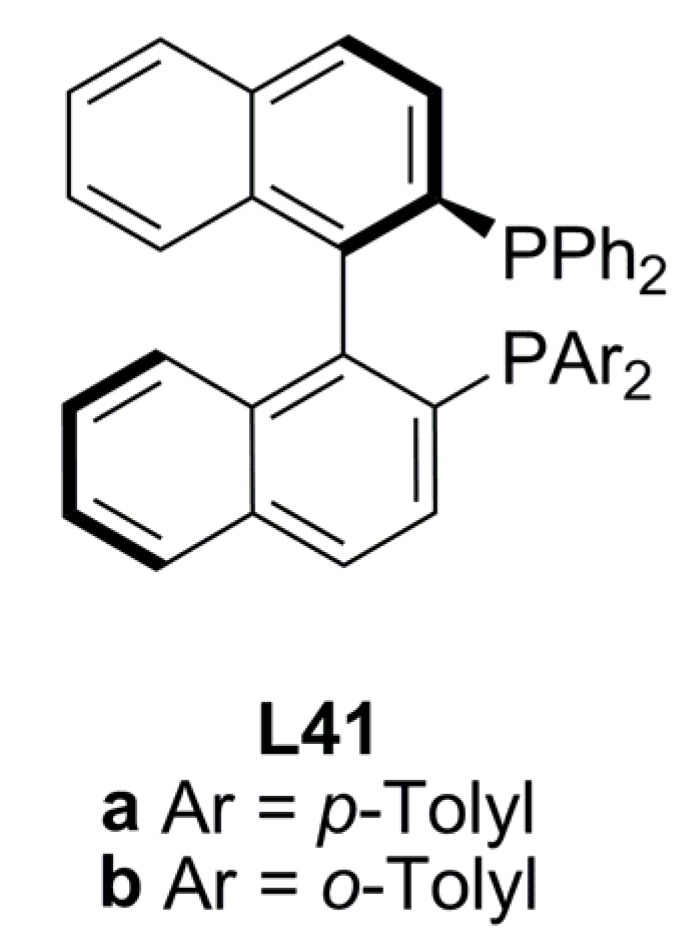
BINAPP’ ligands.

Asymmetric palladium-catalysed alkylation of *rac*-**1a** with carbon nucleophiles (Nu = dimethyl malonate, dimethyl methylmalonate, acetyl acetone, and diethyl acetamidomalonate) proceeded in water in the presence of surfactant cetyltrimethylammonium hydrosulfate (CTAHSO_4_), K_2_CO_3_ and (*R*)-BINAP (**L34**), affording enantioselectivities up to 91%. The efficiency of the catalyst was slightly better in water in the presence of surfactant. The increment of the activity was also observed due to a micellar effect. The alkylation was extended to other allylic acetates, but no micellar effect was detected because the high solubility in water of these allylic substrates compared to *rac*-**1a**, resulting in low values of ee [93]. 

Chiral atropoisomeric bipyrimidinyl diphosphines, **L42**, have been successfully applied in palladium-catalysed asymmetric allylic substitutions. The ligands obtained in high enantiomeric purity by simple synthetic methods were evaluated in the reaction *rac*-**1a** [Equation (1), Scheme 2] and the BSA/base nature, Pd/substrate ratio and temperature were optimized. The catalysts were very active (TOF up to 66 h^-1^) providing 95-81% ee of (*S*)-**2a**. BSA/NaOAc permitted to obtain the best catalytic results compared with others BSA/salt combinations (LiOAc, KOAc and Cs_2_CO_3_) [94]. 

**Figure 18:**
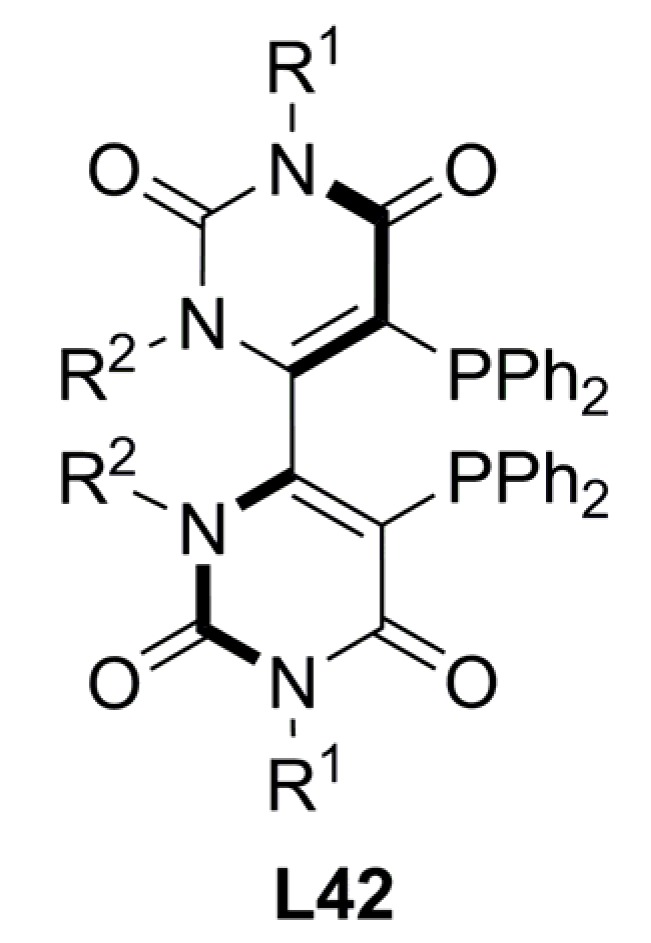
Chiral atropoisomeric bipyrimidinyl diphosphine.

**Figure 19:**
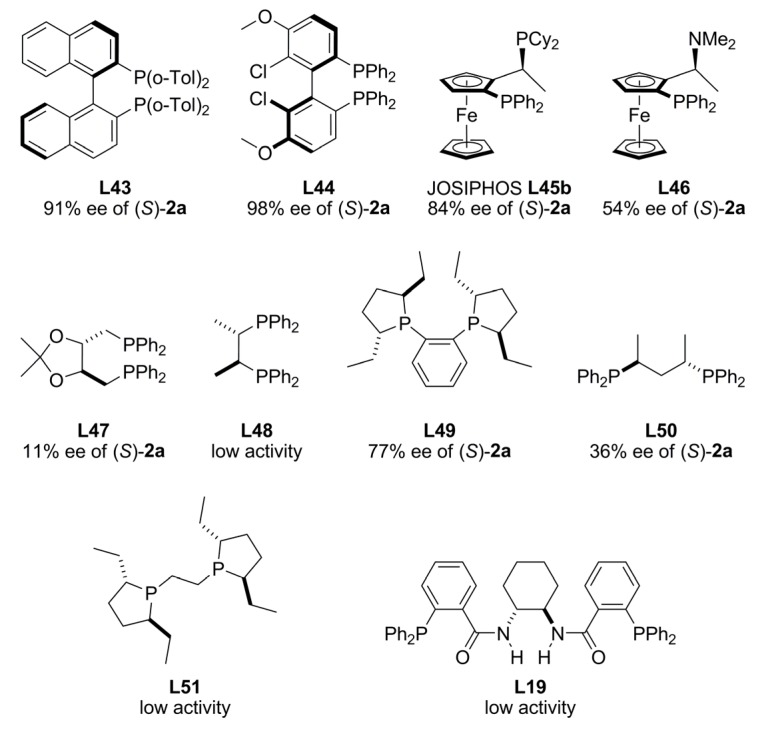
Pd-catalysed allylic alkylation of *rac*-**1a** using ZnEt_2_.

To avoid the use of strong bases, diethyl zinc was recently tested in the enantioselective palladium-catalysed allylic alkylation of *rac*-**1a**. Zinc enolate was generated from direct reaction between diethyl zinc and dimethyl malonate providing up to 99% ee of (*R*)-**2a** for the alkylated compound when Pd/**L34** was used as catalyst, though, this system showed low activity. In THF at reflux, the activity was improved while the enantioselectivity was slightly diminished [97% ee of (R)-2a]. Several known chiral ligands containing stereogenic centres, axes or planes and their combination were examined under these optimized conditions in AAA (Figure 19). 

Ligands with axial chirality furnished higher chiral inductions compared to those with central and/or planar chirality. Interestingly, Trost ligand (*R,R*)-**L19 **and other effective chiral controllers as (*R*,*R*)-Et-DuPHOS (**L49**) and (*R*,*R*)-Et-BPE (**L51**), showed very low activity under these conditions. The combination of (*R*)-BINAP (**L34**) as chiral ligand and ZnEt_2_ to generate the nucleophile was assessed in alkylation of *rac*-**1a** with different carbon nucleophiles giving 97-78% ee. The same excellent performance was observed in the alkylation of racemic 1,3-dialkylallyl acetates with dimethyl malonate [95]. 

**Figure 20:**
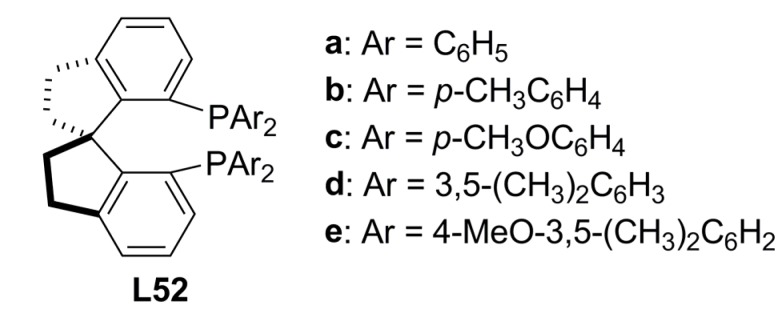
Spiro-diphosphines.

A new type of axially chiral diphosphine ligands, **L52 **(Figure 20), based on the chiral 1,1’-spiro-biindane scaffold have been developed for asymmetric catalysis [96] and their use in palladium-catalysed allylic alkylation have been reported by Zhou and co-workers [97]*.* The results revealed that **L52** ligands are effective in the Pd-catalysed AAA of *rac*-**1a** with dimethyl malonate and related nucleophiles providing the alkylation products in high ee’s (up to 99.1%), with ligand **L52e** being the most selective. It was observed that ligands bearing electron-donating or sterically hindered groups on the P-phenyl rings of the phosphine provided higher enantioselectivities. For instance, ligand **L52e** has 4-methoxy and 3,5-dimethyl groups on the P-phenyl rings of the phosphine.

### 3.3. Diphosphines with planar chirality

The most important chiral ligands bearing stereogenic planes are based on ferrocene skeleton and have been widely used in asymmetric metal-catalysis [56,98,99,100]. The use of ferrocene as scaffold allows tuning the electronic and steric properties of the ligand by simple derivatization. The 1,2-disubstituted- ferrocene ligands display planar chirality combined or not with other chiral elements such as stereogenic centres or axes. JOSIPHOS **L45** is an example of a successful chiral inductor containing planar and central chirality (Figure 19) [91]. The 1,1’-disubstitued ferrocenes have no planar chirality on the metallocene backbone, however, upon coordination to a metal can generate axial chirality [101]. There are few examples of diphosphines exhibiting only planar chirality, among them are PHANEPHOS **L53** [102], 1,1’- and 1,2-bis(phosphino)ferrocenes, **L54** [103,104] and **L55 **[105], phosphaferrocene **L56 **[106] and **L57 **[107]. 

**Figure 21:**
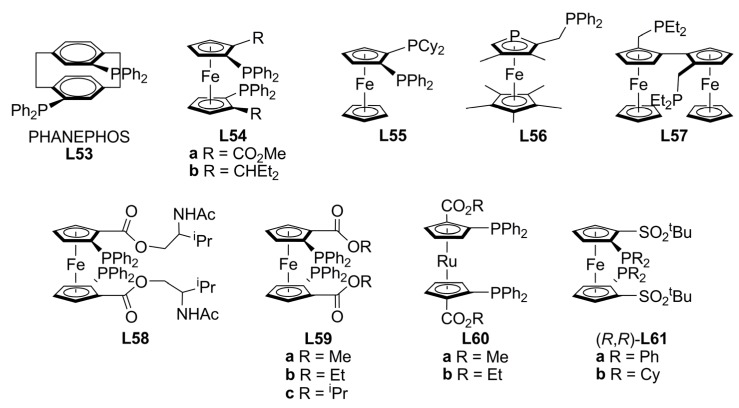
Diphosphines with planar chirality.

Ikeda and co-workers showed that *C_2_*-symmetric ferrocene ligands with planar chirality can produce an excellent chiral environment to obtain high enantioselectivities for palladium-catalysed allylic alkylation. 1,1’-bis(diphenylphosphino)-2,2’-disubstituted-ferrocene ligands **L58-L59 **(Figure 21) afforded 86-92% ee in the alkylation of *rac*-**1a** and up to 83% ee in the alkylation of cyclic substrate *rac*-**3b** with dimethyl malonate. The best catalytic system, Pd/**L59a**, possessing only planar chirality, was also proved in the alkylation of *rac*-**1b** but only 12% ee was achieved [108].

Ruthenocene analogues, **L60**, were recently synthesised and screened in palladium-catalysed asymmetric allylic substitutions. The novel *C_2_*-symmetric diphosphine ligands bearing only the planar chirality of ruthenocene, 1,1’-bis-(diphenylphosphino)-2,2’-disubstituted-ruthenocenes (**L60**, Figure 21) showed higher catalytic activity than their ferrocene analogues **L59** in the alkylation of *rac*-**1a** by dimethyl malonate. Asymmetric inductions up to 95.7 % ee of (*S*)-**2a** were attained (-25 °C) with **L60a**. Ligands **L60b** have also proved to be effective chiral ligands in palladium catalysed asymmetric allylic substitutions of *rac*-**3b** affording 80% ee of (*R*)-**4b** in high conversions. Compared to the corresponding ferrocene ligands, lower temperature conditions (-25°C) were needed in order to achieve similar enantioselectivities [109]. From the solid state structures of the palladium dichloride complexes containing **L59 **or **L60**, it was found that the twist angle of the two Cp rings in the complexes clearly had an effect on enantioselectivity. As a result of the metal nature of the metallocene framework, the distance between the Cp groups was shorter for ruthenocene and the twist angle was larger compared to ferrocene. For the palladium catalyzed AAA of *rac*-**1a**, **L60a** with larger twist angle (23.63°) showed higher enantioselectivity, while an opposite trend was observed for substrate *rac*-**3b**, (**L60b** 16.05°). 

Following a similar procedure, ferrocenyl bisphosphine ligands **L61** (Figure 21) have been prepared and tested in the allylic alkylation of *rac*-**1a** with dimethyl malonate as nucleophile under Trost’s basic conditions. The catalytic system Pd/**L61** produced excellent asymmetric inductions [98% ee of (S)-2a] in high yields (up to 98%). A dramatic increment in enantioselectivity was observed when the additive, metal acetate, was changed from Li^+^ to Cs^+^ [110].

**Figure 22:**
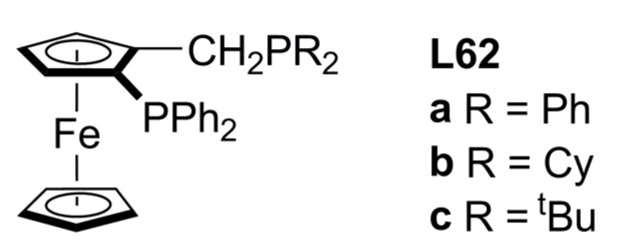
JOSIPHOS-related diphosphines

The alkylation of *rac*-**1a** by dimethyl malonate using 1,2-bis(phosphino)ferrocenes (**L62a-c**, Figure 22) leads to ee’s of 12, 62 and 83% of (*S*)-**2a** respectively [111]. Comparing with the ee’s obtained with the structurally related ligand JOSIPHOS **L45 **[**a**: 81% of (*S*)-**2a** and **b**: 93% of (*S*)-**2a**] [91] it is possible to conclude that an additional stereogenic element improves the asymmetric performance of this type of ligands. 

**Figure 23:**
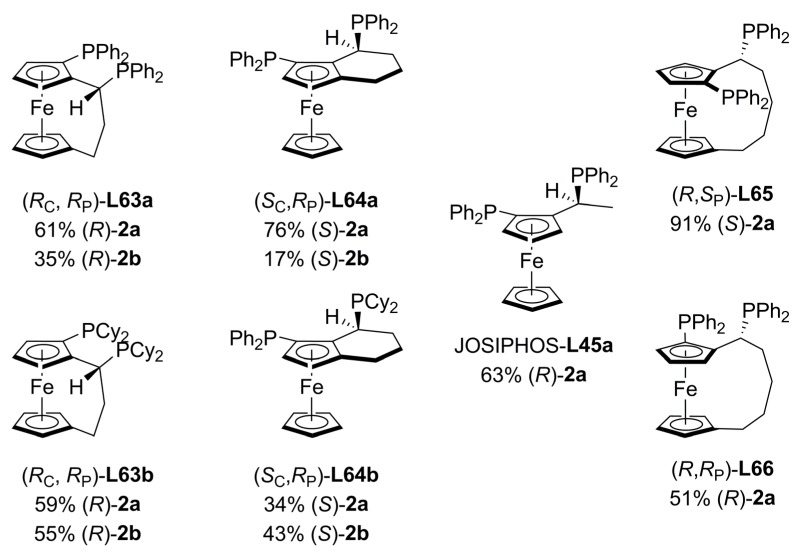
Annularly bridged ferrocenyl diphosphines.

Sets of hetero- and homoannularly bridged ferrocenyl diphosphine ligands, **L63 **and **L64** (Figure 23), were tested in palladium-catalysed allylic alkylation of *rac*-**1** with dimethyl malonate as nucleophile affording enantioselectivities from low to moderate. In the allylic alkylations the maximum ee values obtained were 76% from *rac*-**1a **and 55% from *rac-***1b**. The comparisons were done in two ways: a) functional groups or b) identical backbone. However, the enantioselectivity does not depend on changes on the functional groups or changes on the ligand backbone whatsoever. In addition, the NMR ratios of M-isomer/W-isomer palladium complexes with these ligands do not correlate properly with the enantioselectivities observed, which can be rationalized in terms of nucleophilic attacks to both allylic terminal carbons at different rates [112].

While asymmetric inductions of **L63a** and **L64a** are comparable to JOSIPHOS **L45a** [91], the enantioselectivity of JOSIPHOS **L45b** is much better than those of the related **L63b** and **L64b** annularly bridged ligands. In addition, asymmetric allylic alkylation of *rac*-**1b** with dimethyl malonate showed that central chirality in this kind of complexes controls the configuration of the product.

New [5]ferrocenophane-based diphosphine ligands (**L65 **and **L66 **in Figure 23), having planar and central chirality, were prepared and successfully applied to Pd-catalysed allylic alkylation. Enantioselectivities up to 91% ee of (*S*)-**2a** were obtained using ligand (*R*,*S_P_*)-**L65** in the allylic alkylation of *rac*-**1a** with dimethyl malonate. In contrast, the mismatched ligand (*R*,*R_P_*)-**L66** afforded only 51% (*S*)-**2a** of ee in the same reaction [113].

**Figure 24:**
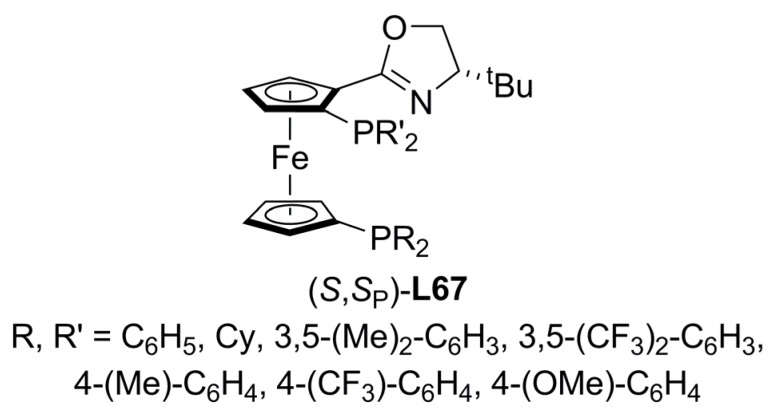
Ferrocenyloxazoline diphosphines.

Ferrocenyloxazoline diphosphines containing both, planar and central chirality (Figure 24) have been screened in the alkylation of *rac*-**1a** [Equation (1), Scheme 2]. Enantioselectivities up to 91.5% ee of (*S*)-**2a **and high yields were attained. The best asymmetric induction was preformed by **L67** substituted with an electronwithdrawing group (R’= 3,5-(CF_3_)_2_-C_6_H_3_), whereas ligands with electrodonor groups (R’=3,5-(CH_3_)_2_-C_6_H_3_) gave poorer enantioselectivities. Since the coordination of **L67** (R=R’=C_6_H_5_) to Pd{1,3-diphenyl-η^3^-allyl} moiety was confirmed, the behaviour exhibited for these ligands was explained in terms of the different *trans*-effect of the phosphorous atoms as well as the steric effect of the oxazoline group to favour a M-type conformation of the catalytically active species [114].

**Figure 25:**
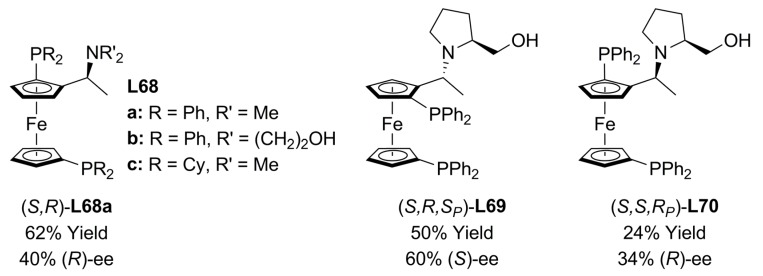
Ferrocenylamine diphosphines.

Chiral ferrocenylphosphines containing α-(2-hydroxyethylamino) side chains (**L68a**, **L69** and **L70**, Figure 25) have proved to be more effective asymmetric inductors than other diphosphines in palladium-catalysed asymmetric allylic alkylation of *rac*-**1a** with NaCH(COMe)_2_ [71-90% ee of (S)-2a] [115]. When chiral ferrocene aminoalcohols, **L69** with three (central–central–planar) stereogenic elements were evaluated in the above mentioned reaction in THF employing K_2_CO_3_ as base, low yields and modest-to-low enantiomeric excesses were obtained. Better asymmetric inductions, up to 73% ee of (*S*)-**2a**, were achieved using 2,5-pentanedione sodium salt as nucleophile [116]. 

The same AAA reaction [Equation (1), Scheme 2] was also investigated by Toma in the ionic liquid [1-butyl-3-methylimidazolium][PF_6_]. Three chiral ferrocenylphosphine ligands containing planar chirality plus central chirality (*S,R*)-**L68a**, (*R,S*)-**L68b** and (*R,S*)-**L68c** were used as chiral inductors and their behaviour was compared to that showed by (*R*)-BINAP, **L34**. It was found that in the presence of K_2_CO_3_ Pd/(*R,S*)-**L68b** [74% ee of (S)-2a] was slightly better chiral inductor than Pd/(*S,R*)-**L68a** system [68% ee of (S)-2a]. The yield of the alkylated product was low in both cases and a decrement of the enantioselectivity was observed in all recycling experiments [117]. Similar performances were displayed by Pd/(*R,S*)-**L68c** and Pd/**L34** when BSA/KOAc was used as base in the catalytic reaction [Equation (1), Scheme 2]. The best ee of the alkylated product [74% ee of (S)-2a] was achieved with **L34** with a TOF value of 2.6 h^-1^. Attempts to re-use the catalyst led to loss of both activity and selectivity [118]. 

**Figure 26:**
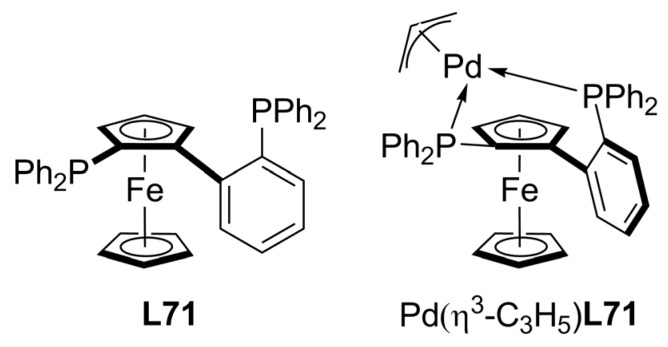
Knochel’s novel diphosphine ligand.

Particularly interesting is the chiral diphosphine with planar chirality **L71** that generates, upon coordination to a metal centre, a new chiral axis (Figure 26). Since the phosphorous donor atoms and the steric fence of bulky ferrocenyl unit, only a single stereoisomer is obtained creating a new stereogenic axis in the metal complex. Thus, in addition to the planar chirality, this ligand bears intrinsic axial chirality. Allylic substitution of *rac*-**1a** with dimethyl malonate in the presence of [Pd(η^3^-C_3_H_5_)Cl]_2_ and the chiral ligand **L71** produced (*R*)-**2a** in high yield and 92–98% ee depending on the temperature. The catalytic system was also very effective in the alkylation of racemic 1,3,3-triphenylprop-2-enyl acetate with dimethyl malonate affording one regioisomer for alkylated product with 94% ee in high yield [119]. The mode of complexation of **L71** was verified in the crystal structure of [Pd(η^3^-C_3_H_5_)**L71**]ClO_4_, demonstrating that the ferrocenyl moiety efficiently shields the bottom side and the back side, directing nucleophilic attack towards the formation of only one enantiomer.

**Figure 27:**
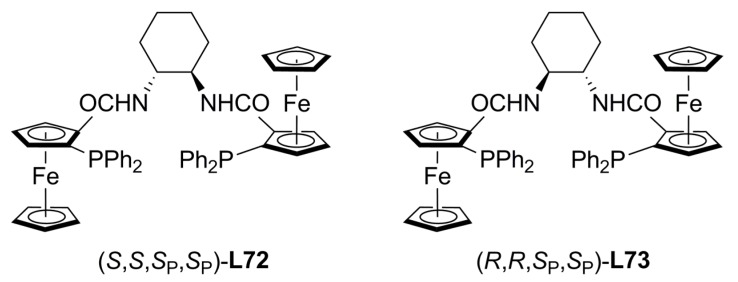
Ferrocenylphosphines analogous to TSL.

In a different strategy Hoe and Dai used the well-known Trost’s chiral pocket concept to design new ferrocene-based ligands, **L72** and **L73** (Figure 27) containing two stereogenic centres and two stereogenic planes. Enantioselectivities up to 95% and 66%ee were obtained using (*R*,*R*,*S_P_*,*S_P_*)-**L72**·2H_2_O and (*R*,*R*,*S_P_*,*S_P_*)-**L73** respectively, in the palladium-catalysed alkylation of allylcarbonates (2-methyl-3-methylcarbonate-1-propene) although no explanation of the effect of two water molecules has been given at the moment [120]. Asymmetric alkylation of iminoesters **36** were also investigated with these ligands [121]. High conversions and low-to-modest enantioselectivities were achieved although ligand **L72** was a more effective chiral inductor, 57.4 % ee of **2a**-(+), than **L73**, 3.8 % ee of **2a**-(+), and other diphosphines such as (*S*)-BINAP, ferrocenylphosphineoxazoline, ferrocenyl-phosphinethioether and cyclohexyl Trost’s ligand **L19**.

**Table 5:** Palladium-catalysed asymmetric allylation of different aminoesters with ligand **L72**.

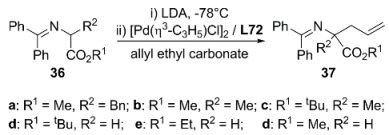		
Substrate	Yield %	%ee
**36a**	87	57
**36b**	93	54
**36b**	92	71
**36c**	95	75
**36d**	94	57
**36e**	93	52
**36f**	92	25

## 4. Miscellaneous Ligands in Asymmetric Allylic Alkylation Reactions

**Figure 28:**
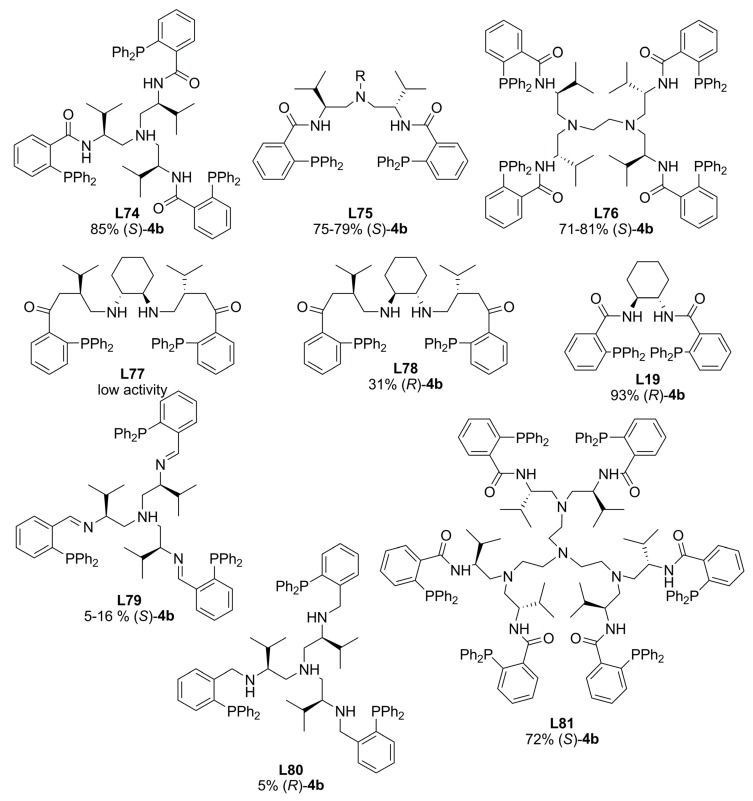
Multidentate ligands.

**Figure 29:**
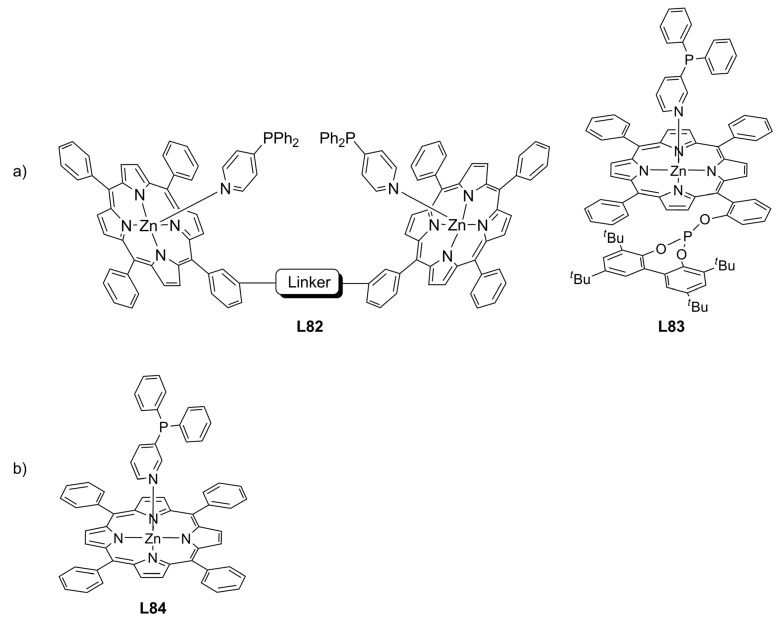
a) Diphosphine SUPRAphos b) Monophosphine SUPRAphos.

Modular multidentate phosphines containing chiral pockets analogues to TSL were synthesised from chiral multidentate amines and 2-(diphenylphosphino) benzaldehyde (Figure 28). In order to investigate the effect of the structure of nitrogen linkers, *C_3_*-symmetric ligands **L79** and **L80** were also prepared. These ligands were evaluated in the alkylation of a number of cyclohexen-3-yl and cyclopenten-3-yl carbonates with dimethyl malonate catalysed by palladium. In the case of *rac*-cyclohexen-2-yl methylcarbonate, TOF up to 240 h^-1 ^and ee’s of 85-72% of (*S*)-alkylated product were achieved employing 2.5% mol Pd with ligands **L74**, **L76** and **L81**. Slightly improvements on the asymmetric induction were obtained with lower palladium loadings (1.25 % mol). The enantioselectivity attained with Trost ligand **L19** was the highest for this reaction [93% (R)] although the catalytic system showed lower activity than most of multidentate ligands. In the case of *rac*-cyclopenten-2-yl ethyl carbonate, **L74**, **L76** and **L81 **gave poor enantioselectivities, 31, 18 and 53% ee of (*S*)-product respectively, at high rates. No study about the metal coordination has been done to correlate the nature of donor atoms and the linker structure on the activity and selectivity of this kind of multidentate ligands [122].

van Leeuwen and Reek have developed a new family of compounds based on the interaction of two monodentate ligands with a zinc-porphyrin template to generate a bidentate ligand (**L82** and **L83**, Figure 29a). This approach is well-known as SUPRAphos concept and it can be applied to form libraries of homo- and hetero-bidentate ligands as well as monodentate ligands **L84** [123,124,125,126].

**Figure 30:**
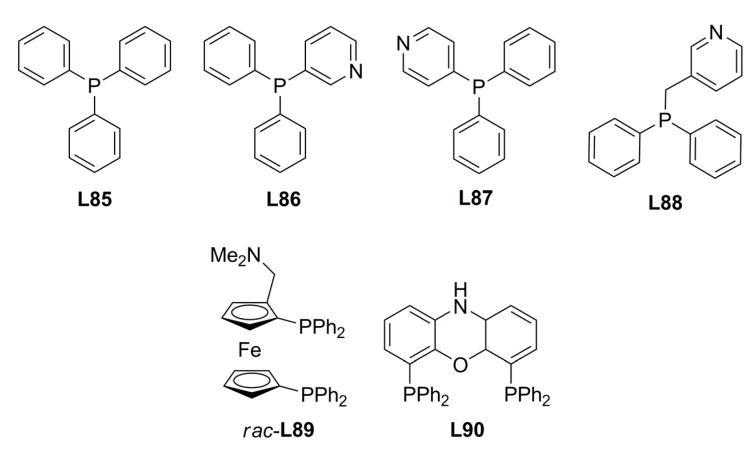
Phosphines for supramolecular strategies.

**Figure 31:**
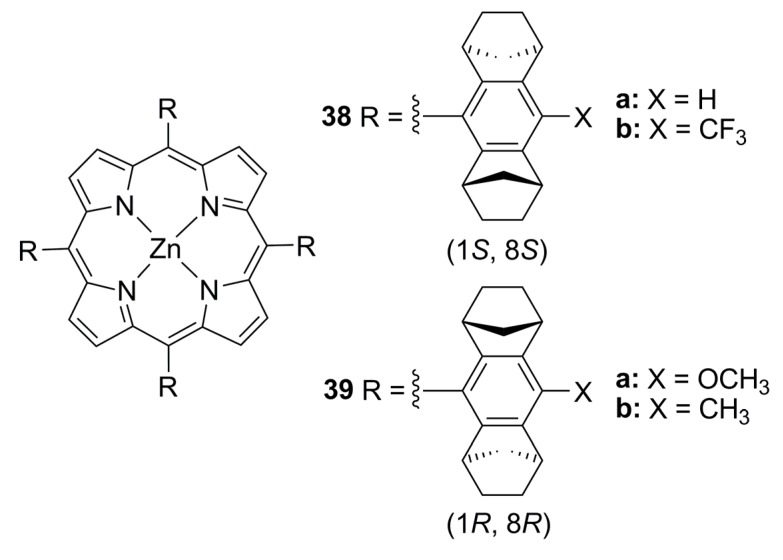
Chiral zinc-porphyrin complexes.

Recently, non-chiral phosphine compounds (**L85**-**L88**, Figure 30) and chiral zinc-porphyrin complexes (Figure 31) were assembled and screened in the palladium-catalysed AAA of *rac*-**1a**, affording low ee’s. Better results were attained when racemic (**L89**) and nonchiral (**L90**) diphosphines were used to bind the chiral zinc-porphyrin template (**38** or **39**) [18% and 12% ee (S), respectively] [127].

## 4. Conclusions

In summary, the Pd-catalysed formation of enantiomerically pure compounds in the presence of chiral phosphine ligands has been unambiguously accomplished, to the point of gaining wide application in the synthesis of complicated organic molecules. In spite of all the efforts to design a ligand that could fit any C-C bond formation, the complicated suitable combination of substrate and nucleophile encourages chemists to design ligands and test them with a variety of reactants. Moreover, the reaction conditions, particularly nucleophile generation, should be taken into account to achieve high catalyst performance. The present review shows successful application of mono- and diphosphines containing different stereogenic elements in AAA, albeit the main body of ligands used to date corresponds to heterodonors also combined with phosphorous-donor atoms. Therefore, the use of chiral phosphines as asymmetric inductors in AAA is a promising area in chemistry to be investigated in the future.
